# Gene expression studies of a human monocyte cell line identify dissimilarities between differently manufactured glatiramoids

**DOI:** 10.1038/srep10191

**Published:** 2015-05-22

**Authors:** Sarah Kolitz, Tal Hasson, Fadi Towfic, Jason M. Funt, Shlomo Bakshi, Kevin D. Fowler, Daphna Laifenfeld, Augusto Grinspan, Maxim N. Artyomov, Tal Birnberg, Rivka Schwartz, Arthur Komlosh, Liat Hayardeny, David Ladkani, Michael R. Hayden, Benjamin Zeskind, Iris Grossman

**Affiliations:** 1 Immuneering Corporation, Cambridge, MA, USA; 2 Teva Pharmaceutical Industries, Petach Tikva, Israel

## Abstract

Glatiramer Acetate (GA) has provided safe and effective treatment for multiple sclerosis (MS) patients for two decades. It acts as an antigen, yet the precise mechanism of action remains to be fully elucidated, and no validated pharmacokinetic or pharmacodynamic biomarkers exist. In order to better characterize GA’s biological impact, genome-wide expression studies were conducted with a human monocyte (THP-1) cell line. Consistent with previous literature, branded GA upregulated anti-inflammatory markers (e.g. *IL10*), and modulated multiple immune-related pathways. Despite some similarities, significant differences were observed between expression profiles induced by branded GA and Probioglat, a differently-manufactured glatiramoid purported to be a generic GA. Key results were verified using qRT-PCR. Genes (e.g. *CCL5,* adj. p < 4.1 × 10^−5^) critically involved in pro-inflammatory pathways (e.g. response to lipopolysaccharide, adj. p = 8.7 × 10^−4^) were significantly induced by Probioglat compared with branded GA. Key genes were also tested and confirmed at the protein level, and in primary human monocytes. These observations suggest differential biological impact by the two glatiramoids and warrant further investigation.

Glatiramer Acetate (Copaxone®; GA), approved in the US since 1996 for treating relapsing forms of multiple sclerosis (RRMS), has been studied for decades, but its precise mechanism remains to be fully elucidated. GA is a synthetic mixture of polypeptides produced by copolymerization of L-glutamic acid, L-alanine, L-tyrosine, and L-lysine with an average molar fraction of 0.141, 0.427, 0.095, and 0.338, respectively. As a non-biological complex drug (NBCD), GA does not possess a single molecular structure, but is comprised of related, differing structures that cannot be isolated or fully characterized using standard analytical techniques^1^. Without established pharmacokinetic (PK) or pharmacodynamic (PD) biomarkers there is substantial uncertainty in attempts to create a generic and assure that it is safe and effective without conducting a clinical study.

GA is believed to exert its clinical effects largely via bystander suppression. It was designed to mimic the autoantigen myelin basic protein (MBP), which is attacked by the immune system in MS. Following degradation at the injection site, GA is thought to bind MHC Class II molecules on antigen-presenting cells (APCs) to generate GA-specific T-cells, mainly helper-T type 2 (Th2)^2^. GA also induces type-II monocytes, directing differentiation of Th2 and protective regulatory T (Treg) cells^3^,^4^. GA-specific T-cells migrate through the blood-brain barrier (BBB), cross-reacting with similarly structured MBP. This reaction induces secretion of anti-inflammatory cytokines locally, shifting the balance from a pro-inflammatory phenotype (Th1/Th17), to an anti-inflammatory phenotype (Th2/Treg)^2^. GA also promotes production of neurotrophic factors such as BDNF, and induces B-cell activation, which appears necessary for GA response in animal models^5^. GA may also work via additional mechanisms.

APCs are central to the mechanism of action (MOA) of GA, as they are necessary for presentation of GA to T-cells during priming at the periphery, and for introducing GA-specific T cells to auto-antigens in the brain. Additionally, GA induces a shift in monocytes to a type II, anti-inflammatory state, marked by increased production of anti-inflammatory cytokines (e.g. IL-10), and decreased production of pro-inflammatory cytokines (e.g. IL-12)^3^. GA has also been found to decrease expression of inflammatory IL-1beta and increase expression of anti-inflammatory sIL-1Ra in monocytes^6^. GA binding to MHC class II molecules on APCs is critical for its activity^2^, and alleles in the MHC class II molecules, most notably HLA-DRB*1501, are associated with treatment response^7^. The effects of GA treatment on human monocytes are therefore likely central to the drug’s MOA, and significant differences observed in the response of monocytes to branded GA versus differently manufactured glatiramoids may be clinically relevant. For these reasons, we chose the well-characterized THP-1 human monocyte cell line for use as a model system for observing effects of GA treatment. Findings were then tested in primary human monocytes from healthy donors.

It is challenging to evaluate drugs designed as generics for GA due to the insensitivity of standard physicochemical and biochemical tests and the lack of PK and PD biomarkers. Still, certain physicochemical characterizations have shown differences between branded GA and other glatiramoids^1^. The purported generics tested to-date show a certain degree of similarity, as well as some discordance in complex physicochemical tests, indicating that the use of additional orthogonal tests may be required to deduce potential sameness. Indeed, establishing methods to evaluate sameness of NBCDs remains an open research question.

One glatiramoid demonstrating potential differences is Probioglat (Probiomed), a purported generic GA marketed in Mexico as of January 2013. The number of adverse events and relapses tracked by the Teva Patient Support Program in Mexico increased significantly in 2013, when patients’ prescriptions were filled with either GA or Probioglat, relative to 2012, when all patients in the database were taking branded GA only (Supplementary Table 5; Supplementary Figure 2). To compare GA with Probioglat, and further elucidate GA’s MOA, we measured gene-expression profiles induced by each in the THP-1 human monocyte cell line, using a genome-wide microarray with 47,000 + probesets.

## Results

### GA mechanism of action

To gain insight into GA’s MOA, the effect of GA treatment on THP-1 human monocytes was examined, since as discussed above, antigen-presenting cells in general, including monocytes in particular, have been shown to be involved in GA treatment effects^3^,^4^,^6^,^8^,^9^. In particular, the THP-1 cell line has been used to examine GA effects^10^. In addition, genes associated with monocytes in particular have previously been shown to be differentially expressed following treatment with a purported generic marketed by Natco in India, as compared with Copaxone^11^.

mRNA expression levels were compared between GA and control (mannitol) tested with 6 sample replicates for each of 4 batches of GA and for mannitol, using LIMMA^12^ (Methods). Many genes were modulated significantly (FDR-adjusted p-value < 0.05) at each timepoint by treatment with branded GA (Table 1; Supplementary Table 1 lists top modulated probesets). For example, at 6 hours of GA treatment, 2824 genes were significantly increased in expression (here termed upregulated) by FDR-adjusted p-value < 0.05 (3511 genes by nominal p-value < 0.05) and 4066 genes were significantly decreased in expression (here termed downregulated) by FDR-adjusted p-value < 0.05 (4909 genes by nominal p-value < 0.05). Fewer genes were significantly modulated as treatment time increased, with approximately half as many modulated at 12 hours, and approximately one-quarter at 24 hours (Table 1). We chose 6 h for initial downstream analysis since this timepoint reflects the greatest impact of treatment. The fact that GA levels persist in cell culture medium over 24 h (Supplementary Figure 1) indicates that this observation is unlikely to reflect decreased drug concentration, but rather that GA impacts expression most pronouncedly at 6 h. The use of this early timepoint may also be biologically relevant given that GA is thought to be rapidly degraded at the injection site, eventually without measurable blood levels^13^,^14^.

**Figure 1:**
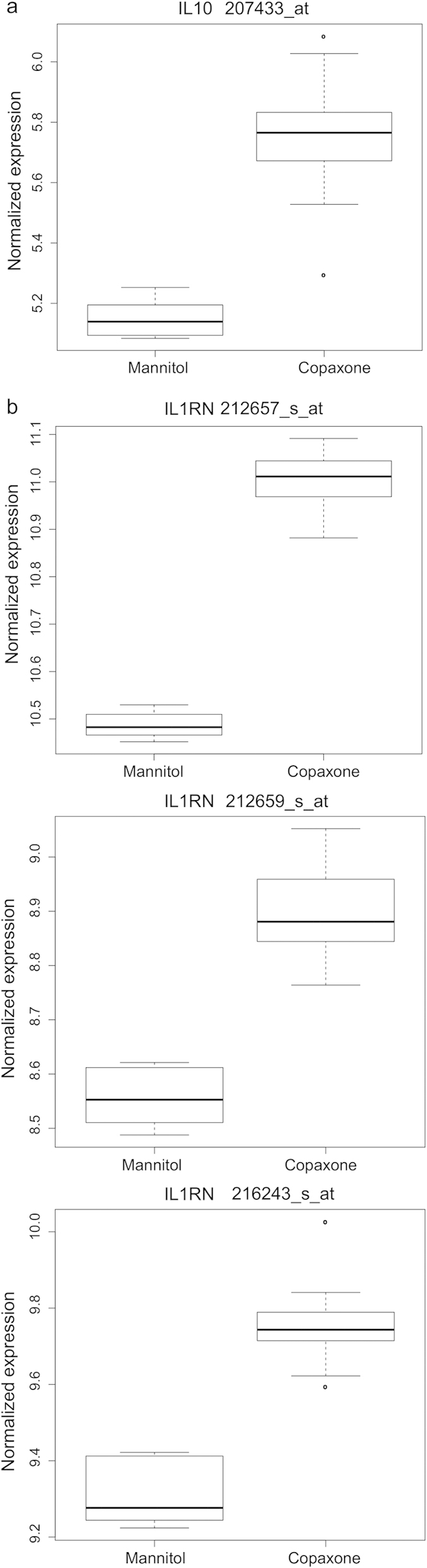
GA treatment increases expression of *IL10* and *IL1RN* (**a**) Increased expression of *IL10* with GA treatment at 6 hours for the single *IL10* probeset on the array (207433_at), FDR-adjusted p < 3.1e-9. (**b**) Increased expression of *IL1RN* following GA treatment at 6 hours for multiple probesets (adjusted p values as provided in text).

The differentially-expressed genes included several anti-inflammatory genes. For instance, *IL10*, the gene encoding the anti-inflammatory cytokine IL-10, was increased in expression at the 6h timepoint (FDR-adjusted p-value 3.1e-9; fold change (FC) 1.52; Fig. 1a). Expression of *IL1RN*, encoding IL-1ra, which inhibits the activities of the pro-inflammatory cytokines IL-1a and IL-1b, was increased at all three timepoints. Figure 1b shows the 6h timepoint for all present probesets (FDR-adjusted p-values 6.7e-16, 1.7e-10 and 1.2e–9, and FC 1.43, 1.35, and 1.26, respectively).

**Figure 2:**
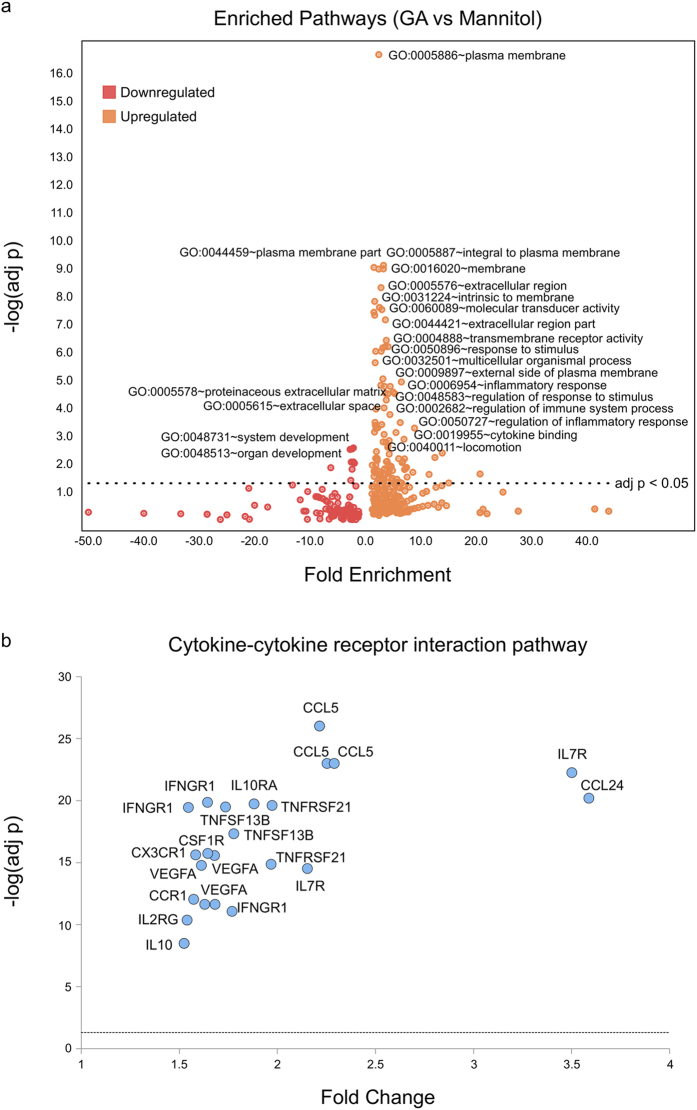
Pathway enrichment among top genes modulated by GA (**a**) Pathways enriched among top genes modulated by GA at 6 hours (restricted to fold-change and adjusted p value filters of 1.5 and 1e-5, respectively). The volcano plot shows –log(adjusted p value) for the enrichment plotted versus the fold enrichment score from DAVID for each pathway. (**b**) Probesets for cytokine-cytokine receptor interaction pathway genes significantly modulated by GA at 6 hours (restricted to fold-change and adjusted p value filters of 1.5 and 1e-5, respectively). The volcano plot shows –log(adjusted p value) for differential expression plotted versus the fold change from LIMMA for each probeset.

To determine whether the differentially-expressed genes related to one another in a coordinated fashion, top significantly up- and down-regulated genes were examined for pathway enrichment using DAVID^15^ as described in Methods (Fig. 2a; Supplementary Table 2). The top genes upregulated by GA in the human THP–1 cell line at 6 h of treatment were enriched significantly (Benjamini-corrected p-value < 0.05) for 114 pathways (Supplementary Table 2), including many immune-related pathways. For example, the top upregulated genes in the cytokine-cytokine receptor interaction pathway (hsa04060) are shown in Fig. 2b. Additionally, 9 pathways were significantly enriched among genes downregulated by GA (Supplementary Table 2).

### Gene-expression differences induced by Probioglat versus GA

Differential gene-expression analysis was performed to compare directly between profiles induced by branded GA and by the purported generic glatiramoid, Probioglat. The standard R LIMMA bioconductor package was utilized to measure differentially-expressed probesets across the entire microarray. Many significant differences were observed between GA and Probioglat (Table 2). As expected based on the more extensive response to GA at 6 h, the most differences were observed at the 6 h timepoint. See Supplementary Table 3 for the full list of differentially-expressed probesets at 6h: 138 upregulated, 24 downregulated (126 upregulated, 22 downregulated after presence/absence filtering).

These differences included pro-inflammatory genes showing increased expression with Probioglat versus GA, including *CCL5, CCL2, MMP9, MMP1, CXCL10, CD14, ICAM1* and *BIRC3* (all significant by FDR-adjusted p-value < 0.05, described in the Discussion). Differences were also observed in levels of anti-inflammatory genes. Probioglat downregulated *CISH* and *HSPD1* and upregulated *IL10* and *PRDM1* relative to GA (all significant by FDR-adjusted p-value < 0.05, described in the Discussion).

**Figure 3:**
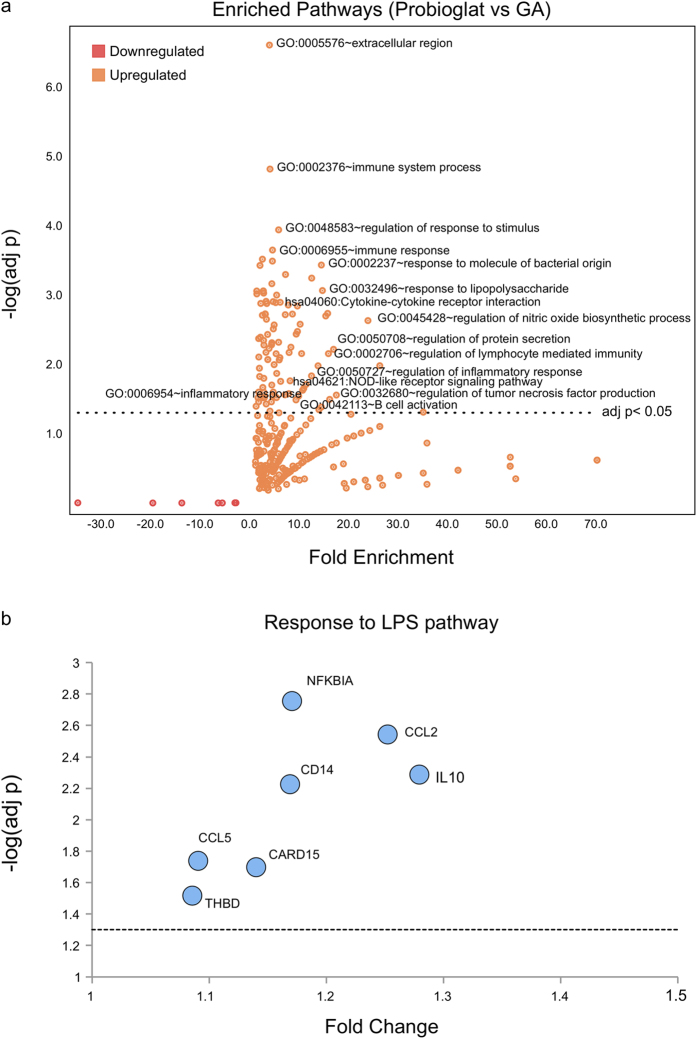
Pathway enrichment for genes upregulated by Probioglat compared with GA (**a**) Pathways enriched among genes upregulated by Probioglat stimulation compared with GA at 6 hours. The volcano plot shows –log(adjusted p value) for the enrichment plotted versus the fold enrichment score from DAVID for each pathway. (**b**) Focus on response to LPS pathway, differentially expressed by Probioglat versus GA at 6 hours. The volcano plot shows –log(adjusted p value) for differential expression plotted versus the fold change from LIMMA for each probeset.

Overall, 106 pathways were enriched significantly (Benjamini-corrected p-value < 0.05) among genes upregulated by Probioglat relative to GA, including general terms like immune system process (GO:0002376) and immune response (GO:0006955) pathways (Benjamini-corrected p-values 1.5e–5 and 3.3e–4, respectively), as well as many other immune-related pathways, such as regulation of lymphocyte mediated immunity (GO:0002706, Benjamini-corrected p-value 0.007) and B cell proliferation (GO:0042100 Benjamini-corrected p-value 0.049) (Fig. 3a; Supplementary Table 4a-b). Multiple significantly enriched pathways were relevant to inflammation, including response to lipopolysaccharide (LPS) (GO:0032496; Fig. 3b), regulation of inflammatory response (GO:0050727), regulation of tumor necrosis factor production (GO:0032680), and NOD-like receptor signaling (hsa04621) (Benjamini-corrected p-values of 8.7e-4, 0.015, 0.028, and 0.027, respectively). No pathways were enriched significantly among genes downregulated by Probioglat versus GA.

### qRT-PCR validates upregulation of pro-inflammatory markers by Probioglat versus GA treatment at 6h

To validate the expression results comparing Probioglat with GA for key inflammation and MS-related genes, two chemokines (*CXCL10*, FDR p-value < 0.0006 with FC 1.46 and *CCL5*, FDR p-value < 0.02 with FC 1.09), two matrix metalloproteinases (*MMP1*, FDR p-value < 0.002 with FC 1.50 and *MMP9*, FDR p-value < 2.8e-6 with FC 1.29) and a non-secreted cell surface marker (*CD9*, FDR p-value < 0.002 with FC 1.15) that is a component of myelin and a marker of myelinogenic progenitor cells^16^ were tested independently by robust qRT-PCR analysis (see Methods). All the genes tested were significantly differentially expressed between Probioglat and GA, as expected based on the microarray analysis (Table 3).

### Protein levels at 24h are consistent with upregulation of pro-inflammatory mRNA markers by Probioglat versus GA treatment at 6h

**Figure 4:**
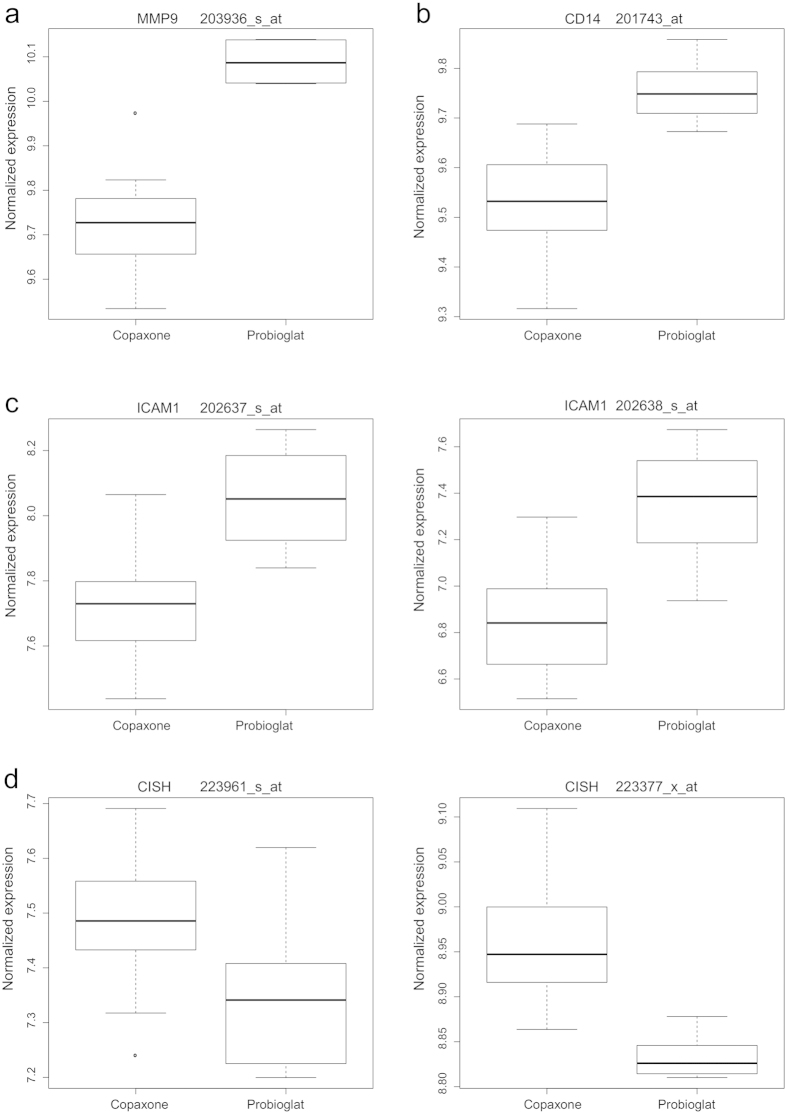
Expression levels of genes differing between Probioglat and GA (**a**) *MMP9* is significantly upregulated following stimulation by Probioglat compared to GA at 6 and 24 hours (FDR-adjusted p values for the single *MMP9* probeset on the chip, 203936_s_at, are 2.74e-6, 0.098, and 0.004 for the 6, 12, and 24 hour timepoints, respectively). (**b**) *CD14* expression is significantly higher with stimulation by Probioglat compared to GA at 6 hours (the single *CD14* probeset on the chip is shown, 201743_at).(**c**) Both present *ICAM1* probesets are significantly upregulated following stimulation by Probioglat compared to GA at 6 hours (A: probeset 202637_s_at; B: probeset 202638_s_at). (**d**) *CISH* is downregulated following stimulation by Probioglat compared to GA at 6 hours (both present probesets are shown, A: probeset 223961_s_at; B: probeset 223377_x_at).

Protein concentration was tested in the same experiment at the 24 h timepoint in order to validate upregulation of pro-inflammatory markers by Probioglat versus GA. Taking into consideration the fact that differences observed at the mRNA level do not necessarily translate to protein concentration differences, and may reflect regulatory processes, a Luminex kit measuring the concentrations of a panel of 45 chemokines and cytokines (in pg/ml) was employed. The Bio-Plex Human Chemokine (Bio Rad kit) and the Luminex Performance Assay (R&D kit) were utilized. Protein concentrations were measured in a single replicate at 24 hours, a timepoint estimated to correspond to the time when the mRNA signals observed at 6 hours may have been translated to protein. Of the five genes tested by qRT-PCR, three were represented on the Luminex panel: *CCL5*, *CXCL10*, and *MMP9*. All three showed higher concentrations in the Probioglat samples than the GA samples (fold changes 1.5, 2.3, and 1.4, respectively; Supplementary Figure 5) consistent with the directionality of the gene expression data (Fig. 4a-c). Two other genes discussed above, *IL10* and *CCL2*, were also present on the Luminex panel and also showed higher concentration levels in Probioglat relative to GA (fold changes 1.8 and 1.3, respectively; Supplementary Figure 5), consistent with the directionality observed at the mRNA level (Fig. 4d,e).

### Key genes upregulated by Probioglat compared to GA were validated in primary human monocytes

**Figure 5:**
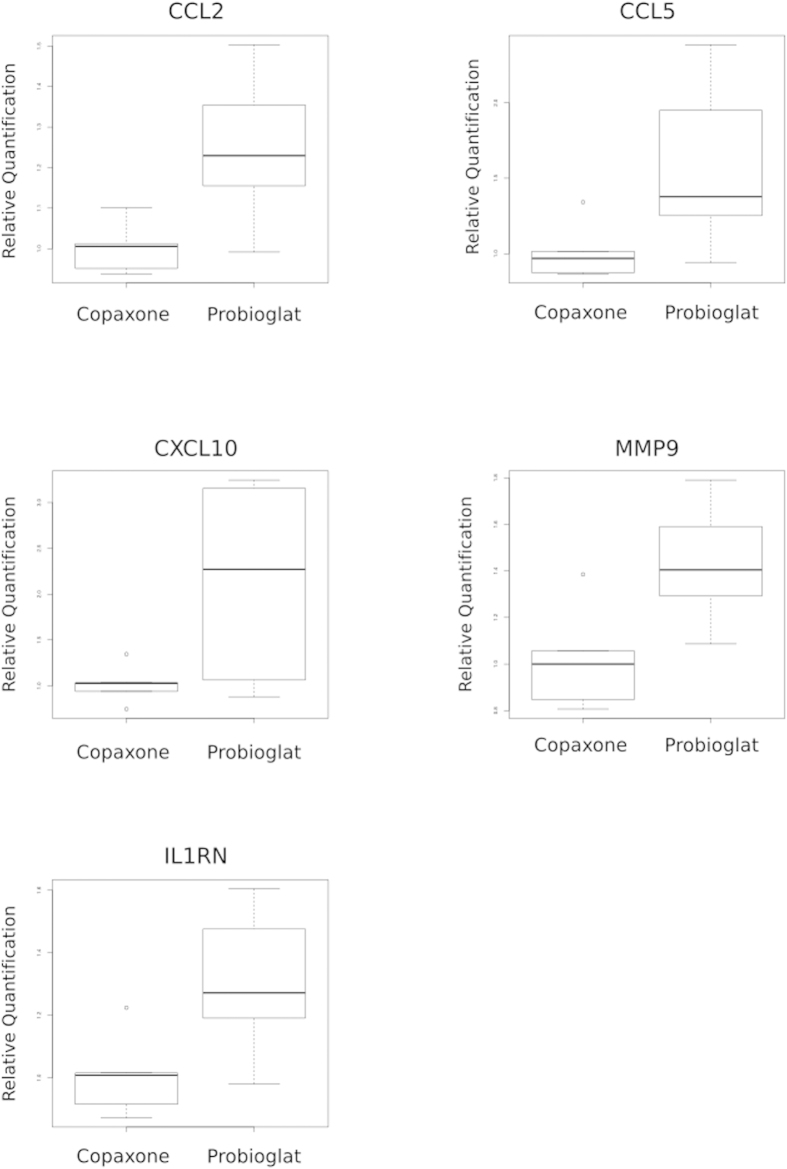
Expression levels of genes differing between Probioglat and GA by qRT-PCR in primary human monocytes. *CCL2* (p < 0.009), *CCL5* (p < 0.029), *CXCL10* (p < 0.020), *MMP9* (p < 0.009), and *IL1RN* (p < 0.013) are expressed more highly under Probioglat stimulation relative to GA stimulation.

While immortalized cell lines are widely utilized in biological research and provide various advantages including uniformity and accessibility, it is important to confirm that the changes introduced by the immortalization process do not alter the key results. Therefore, top findings from the expression data were further tested in primary monocytes from healthy human donors using the sensitive method of qRT-PCR. Nine genes (*CCL2, CCL5, CXCL10, MMP1, MMP9, CD9, ICAM1, IL10, IL1RN*) were chosen for testing based on the findings reported above from the THP-1 monocyte cell line. In primary monocytes from a healthy donor with 6 replicates, the majority of the tested genes exhibited the expected directionality of expression differences between Probioglat and GA. Five of these nine genes passed statistical significance (Fig. 5). These genes included *IL1RN*, and the pro-inflammatory *CCL2, CCL5, CXCL10* and *MMP9* (p values < 0.01 and 0.009, 0.029, 0.02, and 0.009, respectively).

## Discussion

The significant gene-expression changes observed in the human THP-1 cell line due to treatment with branded GA included changes consistent with previous literature (as discussed below), supporting the validity of the chosen model system and current study design for revealing relevant treatment effects.

For example, expression of the anti-inflammatory gene *IL10* was increased at the 6h timepoint, consistent with known GA mechanism in monocytes^3^,^4^,^17^. As discussed above, GA’s anti-inflammatory effect is mediated by secretion of IL-4, IL-10, and other anti-inflammatory cytokines in terms of both T-cells (Th1 to Th2 shift) and monocytes, resulting in a shift from monocyte production of IL-12 to anti-inflammatory IL-10. For example, monocytes from mice treated with GA secreted more IL-10 than monocytes from untreated mice^3^, and monocytes isolated from MS patients treated with GA produced more IL-10 relative to untreated patients.^4^ Additionally, dendritic cells exposed to GA during maturation increased their production of IL-10^17^.

Another anti-inflammatory gene, *IL1RN* (encoding IL-1ra, which inhibits the activities of the pro-inflammatory IL-1a and IL-1b) showed increased expression at all three timepoints. These observations are consistent with work showing that blood levels of soluble IL1-ra protein increased with GA treatment in MS patients and EAE mice, and that levels of soluble IL1-ra increased with GA treatment in human monocytes stimulated with LPS or activated by T-cell contact^6^.

Branded GA significantly modulated many pathways (Supplementary Table 2). At 6h, pathways enriched significantly among upregulated genes included broad categories such as immune response and regulation of immune processes, and more specifically cytokine-cytokine receptor interactions. Other significantly enriched pathways included adhesion, and other pathways with broad relevance to the disease process and/or proposed action of GA. Several of these pathways were also significantly enriched among genes modulated by GA in monocytes from RRMS patients^18^.

Upon comparison of GA with Probioglat, significant gene-expression differences were seen (Table 2). Only one batch of Probioglat was available to compare to the four batches of GA, prohibiting the possibility to study batch-to-batch variability. However, the range of variation defined by the four GA batches represents a range of variation that has been demonstrated to be safe and effective by Copaxone’s clinical trials. The fact that any single batch of Probioglat results in values outside of that range (as illustrated in Supplementary Table 6 and Supplementary Fig. 4), coupled with lack of PK or PD markers to determine equivalence of the two glatiramoids, warrants further investigation. The consistent confirmatory results obtained by single-probeset, pathway and independent qRT-PCR analyses are particularly robust, given the stringent statistical framework employed. It should be noted that fewer genes were significantly modulated by GA relative to Probioglat than by GA relative to mannitol, an observation expected given the intended mimicry of structure between the compounds. Indeed, many genes were modulated in the same direction by both GA and Probioglat versus control, but to differing extents (the case for many genes discussed below, except where noted). Importantly, the significant expression differences observed between GA and Probioglat were seen in genes tied to relevant disease pathoetiology and known MOA of GA. These included a number of genes tied to important immune system functions, particularly inflammation: *CCL5, CCL2, MMP9, MMP1, CXCL10, CD14, ICAM1* and *BIRC3*. Several of these genes are reported in the literature as modulated by GA treatment in patients (as discussed below).

*CCL5* (RANTES), encoding a chemokine thought to attract inflammatory immune cells to the central nervous system (CNS), was upregulated with Probioglat treatment versus GA treatment at 6h (FDR-adjusted p-value 0.018, FC 1.09 in gene-expression analysis; p-value 4e-5, FC 1.12 in qRT-PCR confirmation). Indeed, an antibody blocking CCL5 reduced disease metrics including immune infiltration into the CNS in a viral MS model^19^. Expression of the CCL5 receptor, CCR5, on GA-reactive T-cells from MS patients was shown to be decreased by chronic (1 year) GA treatment^20^. This gene was tested and confirmed to be upregulated with Probioglat treatment relative to GA treatment in primary human monocytes (p < 0.029, FC 1.53). Another pro-inflammatory cytokine gene, *CCL2* (MCP-1), was also expressed significantly more highly with Probioglat versus GA (FDR-adjusted p-value 0.003, FC 1.25). *CCL2* expression was decreased by GA treatment relative to mannitol control, and decreased to a lesser extent by Probioglat relative to mannitol control. CCL2 is thought to recruit inflammatory cells into the CNS in EAE and in MS^21^. This gene was also confirmed to be upregulated with Probioglat relative to GA treatment in primary human monocytes (p < 0.009, FC 1.24).

Expression of *MMP9* (Matrix Metalloproteinase 9) was significantly higher with Probioglat versus GA stimulation at 6 h (FDR-adjusted p-value 2.8e-6, FC 1.29 in gene-expression analysis, Fig. 4a; p-value 0.02, FC 1.24 in qRT-PCR confirmation), and at 24 h (FDR-adjusted p-value 0.004, FC 1.25). The *MMP9* gene was also upregulated with Probioglat relative to GA treatment in primary human monocytes (p < 0.009, FC 1.4). This protein is reported to increase access of immune cells to the CNS by contributing to BBB disruption, and high levels of MMP9 have been associated with MS^22^,^23^,^24^. Elevated MMP9 levels were reported in patients with gadolinium-enhancing lesions versus patients without^25^, and MMP9 has been proposed as a biomarker for both MS diagnosis and progression^26^. GA was reported to inhibit *MMP9* expression in healthy human peripheral blood mononuclear cells (PBMC)^27^.

The level of *MMP1*, another matrix metalloproteinase gene, was increased after Probioglat stimulation compared to GA at 6 h (FDR-adjusted p-value 0.002, FC 1.50 in gene-expression analysis; p-value 0.02, FC 1.25 in qRT-PCR confirmation). Matrix metalloproteinases are known to cleave pro-inflammatory cytokines and chemokines to regulate inflammation^28^. Levels of MMP1 mRNA, and secreted MMP1, were observed to be higher in immature dendritic cells from MS patients versus healthy controls^24^.

Expression of the chemokine gene *CXCL10* was increased by Probioglat compared to GA treatment at 6 h (FDR-adjusted p-value 0.0006, FC 1.46 in gene-expression analysis; p-value 0.0029, FC 2.28 in qRT-PCR confirmation). This finding was confirmed by qRT-PCR in primary human monocytes, where CXCL10 was upregulated by Probioglat relative to Copaxone treatment with p value < 0.02 and fold change of 2.1. CXCL10 level in peripheral fluids was previously shown as associated with host immune response, particularly with regard to Th-1 cells^29^. CXCL10 is involved in recruiting CD8^+^and Th1 CD4^+^effector T-cells to sites of inflammation^30^. A study using monocytes from RRMS patients demonstrated *CXCL10* to be increased by GA therapy within the first two months of treatment^18^.

*CD14* was upregulated in human monocytes stimulated by Probioglat versus GA at 6h (FDR-adjusted p-value 0.006, FC 1.17, Fig. 4b). *CD14* was not modulated by GA treatment versus mannitol control. This marker of monocyte activation enhances inflammatory responses^31^. In complex with LPS-binding protein, CD14 interacts with LPS to help present it to toll-like receptor 4 (TLR4), activating downstream expression of inflammatory genes via NF-kB. CD14 is also a coreceptor for other TLRs, and was demonstrated as required for induction of pro-inflammatory cytokines via TLR7 and TLR9 in mouse and human cells in vitro^32^.

*CARD15* (NOD2), also upregulated by Probioglat versus GA treatment at 6h (FDR-adjusted p-value 0.02, FC 1.14), is another player in the immune response to LPS, where it participates in NF-kB activation. Activation of NOD2 by peptidoglycan induced CNS demyelination in rats^33^, and a SNP in NOD2 was shown to affect the responses of Th2 and Th17 cells to MBP in MS^34^.

*ICAM1* expression was increased by Probioglat versus GA treatment at 6h (FDR-adjusted p-value 0.004, FC 1.41, Fig. 4c). ICAM1 is an adhesion molecule that plays a key role in inflammatory processes by promoting leukocyte adhesion to the endothelium of the vascular wall, and is known to have an important role in inflammatory cell infiltration into the CNS in both EAE and MS^35^. In mice null for ICAM1, T-cells produced significantly less IFNγ and showed decreased infiltration into the spinal cord^36^. In PBMC from RRMS patients, ICAM1 levels were higher versus healthy controls, and chronic GA treatment affected surface ICAM1 levels in multiple immune cell types^37^.

*BIRC3* expression was increased by Probioglat versus GA treatment at 6 h (FDR-adjusted p-value 0.018, FC 1.26). This gene encodes an Inhibitor of Apoptosis Protein (IAP-1), which beyond its role in cell survival affects innate immunity^38^ and inflammation^39^, and may have an immunomodulatory effect in autoimmune demyelination^40^. IAPs including IAP-1 are required for production of pro-inflammatory cytokines via several pathways, including TLR4 activation^41^ and NOD2 activation by TNFα^42^.

The genes upregulated (FDR-adjusted p-value < 0.05) with Probioglat relative to GA treatment at 6 h were found to be enriched significantly (Benjamini-corrected p-value < 0.05) for 106 pathways annotated in the GO (Biological Process, Cellular Component, and Molecular Function) and Kegg databases (Fig. 3a; Supplementary Table 4)^43^,^44^. As mentioned above, these include immune system process (GO:0002376) and immune response (GO:0006955) pathways. Several pathways are relevant to inflammation (e.g., regulation of inflammatory response (GO:0050727) and regulation of tumor necrosis factor production (GO:0032680)). NOD-like receptor signaling (hsa04621) regulates inflammatory and apoptotic responses. The response to LPS pathway (GO:0032496; Fig. 3b) includes the genes *CD14, CCL5, THBD, CARD15, NFKBIA,* and *CCL2,* all increased in expression in Probioglat treatment versus GA at 6 h. This pathway was also significantly enriched among probesets upregulated by GA treatment at 6 h, but with a lower enrichment score (14.8 vs 2.7) and higher p-value (0.00087 vs 0.036). The enrichment induced by Probioglat relative to GA of this prototypical pro-inflammatory pathway warrants further investigation with respect to safety.

Interestingly, about half of the pathways (58 out of 114) significantly enriched (Benjamini-corrected p-value < 0.05) among genes upregulated by GA treatment versus mannitol control at 6 h were also significantly enriched among genes upregulated by Probioglat relative to GA treatment. This indicates that many of the effects of GA treatment were accomplished to a differing extent by Probioglat. What this would imply for efficacy in patients is unclear, and would need to be evaluated in the appropriate setting. Additionally, 48 pathways were significantly enriched among genes upregulated by Probioglat relative to GA (and not modulated by GA relative to mannitol control). These include pathways relevant to inflammation, such as regulation of TNF production, NOD-like receptor signaling pathway, and response to molecule of bacterial origin (GO:0002237), and other immune pathways including regulation of lymphocyte mediated immunity (GO:0002706) and B cell proliferation (GO:0042100).

While pro-inflammatory genes and pathways were significantly upregulated by Probioglat relative to GA, several anti-inflammatory genes were differently expressed with Probioglat relative to GA treatment at 6 h.

*HSPD1*, also known as HSP-60 (heat shock 60 kDa protein 1), was decreased in expression by Probioglat relative to GA at 6h (FDR adjusted p-value 0.01, FC -1.31). Zanin-Zhorov *et al.*^45^ showed that HSP60 as well as synthetic peptide derived from HSP60 acted as co-stimulators of anti-inflammatory Tregs through the TLR2 pathway, concluding that HSP60 can downregulate adaptive immune responses by upregulating Tregs.

*CISH*, also known as SOCS (Suppressor of Cytokine Signaling), was expressed at a lower level with Probioglat relative to GA at 6h (FDR adjusted p-value 0.03, FC -1.09; Fig. 4d). A closely-related protein, SOCS3, was shown in myeloid cells to protect from EAE, the mouse model of MS, via deactivating neuroinflammatory responses^46^. A SNP in *SOCS1*, another family member, has been identified as a risk factor for MS^47^.

It should be noted, however, that the anti-inflammatory cytokine gene ***IL10***, known to be relevant to GA MOA, was also expressed at a higher level subsequent to Probioglat treatment relative to GA at 6h (FDR-adjusted p-value 0.005, FC 1.28). The same observation holds for another gene at 6h and 12h, ***PRDM1*** (Blimp1) (FDR-adjusted p-values 0.0006 and 7.7e-6, and FC 1.31 and 1.31, respectively), that when deleted results in inflammatory pathology^48^. Blimp1 is a target of FOXP3 and is needed for production of IL-10 by Tregs, and its expression can also be induced by IL-2 and proinflammatory cytokines in Tregs;^49^ it is not clear what these observations would imply for APCs such as monocytes. It is possible that higher Blimp1 could be an attempted protective response to a higher inflammatory milieu. A statistically significant difference in such a mechanistically relevant gene – in either direction – between two therapeutics intended to be identical presents motivation for further study.

Protein concentrations tested in the same experiment at the 24 h timepoint were consistent with the findings observed at the mRNA level, supporting the reported findings and indicating an inflammation-related biological impact at the protein level. An independent follow-on study in primary human monocytes tested nine inflammation and MS-related genes by qRT-PCR, finding that five of these genes were statistically significantly upregulated by Probioglat relative to GA. These included *IL1RN*, which is relevant to Copaxone mechanism of action. In addition, *CCL2, CCL5, CXCL10,* and *MMP9* were all seen to be upregulated significantly and consistently at both the mRNA and protein level in THP-1 cells, as well as confirmed by qRT-PCR in primary human monocytes. These genes act in pro-inflammatory pathways and have been implicated as relevant to MS susceptibility and severity, as described above.

The complex picture of genomic signatures described here underscores the intricate relationships between immune processes, effects of treatment on the associated pathways and the differing responses of each immune cell type. Consistent with previous evidence from other systems and cell types^11^,^50^, differences are consistently observed between GA and differently-manufactured glatiramoids, although their nature depends on the biological context of the tested model. Further, many of these differences affect molecules relevant to drug MOA and MS disease pathoetiology, particularly relating to inflammatory signatures. Genes significantly upregulated by Probioglat relative to GA were significantly enriched for inflammatory pathways and included key pro-inflammatory genes.

These findings have identified significant differences that warrant further investigation, especially in light of the observed clinical effects of Probioglat’s introduction. The Teva Patient Support Program in Mexico records numbers of adverse events, including during the years 2012 and 2013 (Supplementary Table 5; Supplementary Figure 2), for which a similar number of patients were tracked (1618 patients in 2012; 1552 in 2013). Probioglat was first introduced in January 2013; subsequently, each time a patient’s prescription was filled, it could be with either Probioglat or GA. In 2013 versus 2012 (during which only branded GA was marketed), numbers of adverse events, and relapses specifically, increased more than 3-fold (from 125 to 380) and 7-fold (from 8 to 59), respectively (Supplementary Table 5; Supplementary Figure 2), representing statistically-significant increases (p < 2.2e-29 and p < 3e-11, respectively). The gene-expression differences observed herein warrant careful investigation, through studies comparing GA to candidate generics in meaningful settings, most comprehensively including clinical trials.

## Methods

### Power calculations and experimental design

Using the R package ssize.fdr, power calculations were performed to determine the number of samples needed to detect differentially-expressed genes with a fold-change between treatments of as low as 1.3 with 80% power. Based on these power calculations, the experiment was designed to include six replicates of each condition. The order of sample processing was randomized with respect to treatment in order to avoid creating confounding batch effects.

### Cell line treatment and RNA processing

Cells from a human monocyte cell line (THP-1) were stimulated with either branded GA, purported generics from several manufacturers including Probioglat by Probiomed, or vehicle control (mannitol) for 6, 12, or 24 h. RNA was extracted and expression profiled genome-wide using the Affymetrix U133 Plus 2.0 chip. Four batches of GA and one batch of Probioglat were comparatively tested in six biological replicates each. Microarray data have been deposited to the Gene Expression Omnibus (GEO, GSE68527).

### Batch correction

Correction for batch variation was performed using ComBat^51^, as implemented in the SVA R package^52^. Treatment labels were added as covariates to the batch correction to preserve relevant treatment effects. Principal Component Analysis showed the main effect in PC1 remained due to treatment effects after batch correction (Supplementary Figure 3).

### Differential expression analysis

Differentially-expressed probesets were identified across conditions using linear models for microarray data (LIMMA). For comparing GA and purported generic, comparisons were corrected for mannitol control (i.e., [GA vs mannitol] was compared to [purported generic vs mannitol]). For use in pathway analyses, probesets were filtered by calls of presence on the chip for the relevant samples in the comparison (e.g., to be considered present at a given timepoint, a probeset was required to have an average call of present/marginal across relevant samples at that timepoint). Probesets were mapped to genes using the U133 Plus 2.0 chip annotation from Affymetrix. FDR-adjusted p-values reported for genes represent the lowest FDR-adjusted p-value for present probesets for that gene.

### Pathway enrichment analysis

Upregulated and downregulated genes were analyzed separately for pathway enrichment, using DAVID^15^. Pathway enrichment results were visualized using volcano plots, plotting –log p-values versus enrichment scores. For GA MOA, to obtain top-gene lists of appropriate size (tens-hundreds) for use with DAVID, an absolute-value fold-change cutoff of 1.5 and p-value cutoff of 1e-5 were utilized to obtain gene lists for pathway enrichment at 6 h. For comparisons between GA and Probioglat, upregulated or downregulated genes with FDR-adjusted p-values < 0.05 were used for pathway enrichment.

DAVID runs were conducted May 21, 2014. Please note that the GO databases are updated daily (as noted on the GO site: http://www.geneontology.org/GO.downloads.ontology.shtml) and therefore performing the same enrichments on the same genesets may yield slightly varying results depending on the rundate, as illustrated by the differences between Supplementary Table 4a and 4b (results from runs on differing dates using broader or more restrictive subsets of GO). Thus, pathway p-values may change slightly between runs conducted at different times; the overall picture of enriched pathways, however, is expected to remain consistent.

### qRT-PCR

Key genes identified by differential expression analysis were assayed using qRT-PCR. RNA was utilized from 6 biological samples for each treatment (GA and Probioglat) and 15 technical replicates were performed for each sample (totaling 90 observations/transcript/treatment). To evaluate the data, the 2^−ΔΔCt^ approximation was utilized with GAPDH as reference transcript and vehicle control (mannitol) as calibrator. One-sided t-tests with unequal variance were used to compare expression between treatments.

### Protein Concentrations

THP-1 cells were activated with GA or Probioglat as described above. The supernatant (1.0 mL of cell culture media) was collected at the 24 hour timepoint (to account for the time duration required for translation relative to the 6 hour mRNA data reported herein).

A Luminex assay was utilized to measure the concentrations of a panel of 45 chemokines and cytokines (in pg/ml) using Bio-Plex Human Chemokine (Bio Rad kit) and Luminex Performance Assay (R&D kit). Three of the genes that were found to significantly differ between GA and Probioglat by qRT-PCR (*CXCL10, MMP9*, and *CCL5/RANTES*) had corresponding proteins present in the Luminex panel. In addition, two other genes that were found to differ significantly between GA and Probioglat using the genome-wide microarray mRNA data were also present in the Luminex panel (*CCL2, IL10*).

To calculate the fold change between the protein expression levels with Probioglat and with GA, the values for the four GA batches were averaged together and compared to the value for Probioglat (Probioglat expression level/ average GA expression level).

### Primary Human Monocyte Study

50 mL of blood was obtained from a healthy donor, and CD14 + cells were separated from whole blood using magnetic beads (Miltenyi Biotech). Purity was determined by FACS analysis using the following antibodies: CD14, CD15, CD16, CD45 (BD Biosciences). Into each plate of the 6 well plate 0.5 mL containing 1.0 × 10^6^ cells was added. In addition 0.5 mL of either Copaxone, Probioglat (100 μg/mL) or mannitol (200 μg/mL) was added, total volume in each well was 1mL. Final concentration of Copaxone and Probioglat was 50 μg/mL and mannitol final concentration was 100 μg/mL. Cells were incubated for 6 hours at 37 °C at 5% CO_2_ followed by centrifugation to pellet the cells. The cell pellets were then processed using a Qiagen RNeasy RNA purification protocol.

Expression levels of nine genes were measured using RT-PCR with GAPDH as reference transcript. Analyses reported compared Probioglat samples to GA samples as calibrator. Similar results were obtained when using mannitol as reference (i.e., the same set of genes were significantly upregulated in Probioglat relative to Copaxone). Differences in expression levels were evaluated for significance using one-sided t-tests with unequal variance.

## Author Contributions

Designed experiments: T.H., S.B., DLai, A.G., R.S., A.K., L.H., DLad, M.R.H. and I.G.; Performed experiments: T.H., S.B., A.G., R.S., and T.B.; Analyzed data: S.K., F.T., J.M.F., K.D.F., M.N.A., B.Z., T.H., DLai, M.R.H. and I.G.; Contributed new reagents/analytical tools: S.K., F.T., J.M.F., K.D.F., M.N.A. and B.Z.; Wrote manuscript: S.K., F.T., DLad, A.G., B.Z. and I.G.

## Additional Information

**How to cite this article**: Kolitz, S. *et al.* Gene expression studies of a human monocyte cell line identify dissimilarities between differently manufactured glatiramoids. *Sci. Rep.* **5,** 10191; doi: 10.1038/srep10191 (2015).

## Supplementary Material

Supplementary Information

## Figures and Tables

**Table 1:** Numbers of genes significantly modulated by GA treatment across timepoints.

	**6 hours**		**12 hours**		**24 hours**	
	**Upregulated**	**Downregulated**	**Upregulated**	**Downregulated**	**Upregulated**	**Downregulated**
nominal p<0.05	3511	4909	2377	3430	1410	3724
FDR p<0.05	2824	4066	1308	1810	606	1185
FDR p<1e-5, |FC|>=1.5	257	119	68	10	15	0
FDR p<1e-5, |FC|>=1.3	557	508	210	50	57	6

*FC – Fold Change; FDR – False Discovery Rate correction.

**Table 2:** Dynamic profiles of differentially-expressed genes after stimulation of THP-1 cells by Probioglat versus GA.

**Stimulation time**	**6 hours**		**12 hours**		**24 hours**	
**Significance threshold**	**Genes**	**Probesets**	**Genes**	**Probesets**	**Genes**	**Probesets**
	****	**#**	**#**	**#**	**#**	**#**
Upregulated:						
FDR-adjusted p value<0.05	115	138	5	5	1 (*MMP9*)	1
Nominal p value<0.05	2,597	3,310	1,296	1,560	1,625	1,959
Total modulated (up- and down-regulated):						
FDR-adjusted p value<0.05	136	162	7	7	1 (*MMP9*)	1
Nominal p value<0.05	4,863	6,208	3,051	3,992	2,843	3,486

Numbers of genes and probesets modulated by Probioglat relative to GA.

**Table 3:** Differential expression level of key immunological genes following Probioglat stimulation compared with GA at 6h. Shown are p-values for qPCR results from single-tailed t-tests with unequal variance, and FDR-adjusted p-values from LIMMA comparisons.

**Genes**	**CCL5**		**CD9**		**CXCL10**		**MMP1**		**MMP9**	
**Method**	**FC**	**p value**	**FC**	**p value**	**FC**	**p value**	**FC**	**p value**	**FC**	**p value**
qPCR	1.12	4.05E-05	1.11	0.0004	2.28	0.0029	1.25	0.0201	1.24	0.0168
FDR-adjusted Microarray	1.09	0.02	1.15	0.002	1.46	0.0006	1.5	0.002	1.29	2.80E-06

FC: fold change; qPCR: quantitative RT-PCR; FDR: For the microarray data, since all probesets on the microarray were tested, p values were adjusted using FDR for testing multiple hypotheses.
