# Supplementary material for: Gene expression studies of a human monocyte cell line identify dissimilarities between differently manufactured glatiramoids
Source: Sci Rep. 2015 May 22;5:10191. doi: 10.1038/srep10191 (PMC4441120; doi:10.1038/srep10191)
Supplement: Supplementary Information [file srep10191-s1.pdf]

Supplementary Information for:

Gene expression studies of a human monocyte cell line identify dissimilarities between differently manufactured glatiramoids

Sarah Kolitz,[1*] Tal Hasson,[2*] Fadi Towfic,[1] Jason M. Funt,[1] Shlomo Bakshi,[2] Kevin D. Fowler,[1] Daphna Laifenfeld,[2] Augusto Grinspan,[2] Maxim N. Artyomov,[1] Tal Birnberg,[2] Rivka Schwartz,[2] Arthur Komlosh,[2] Liat Hayardeny,[2] David Ladkani,[2] Michael R. Hayden,[2] Benjamin Zeskind[1,+], Iris Grossman[2,+]

[1]Immuneering Corporation, Cambridge, MA, USA [2]Teva Pharmaceutical Industries, Petach Tikva, Israel [*,+]These authors contributed equally.

SUPPLEMENTARY FIGURE 1

Levels of Copaxone measured over time in cell culture medium:

Copaxone concentration in medium over time remains steady in the range of 44-52 μg/mL over 24 hours.

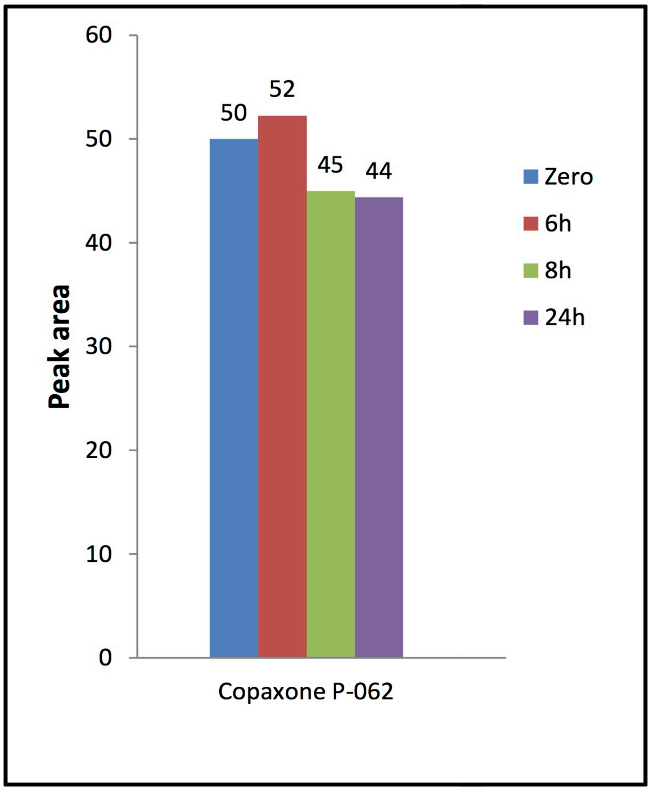

SUPPLEMENTARY FIGURE 2

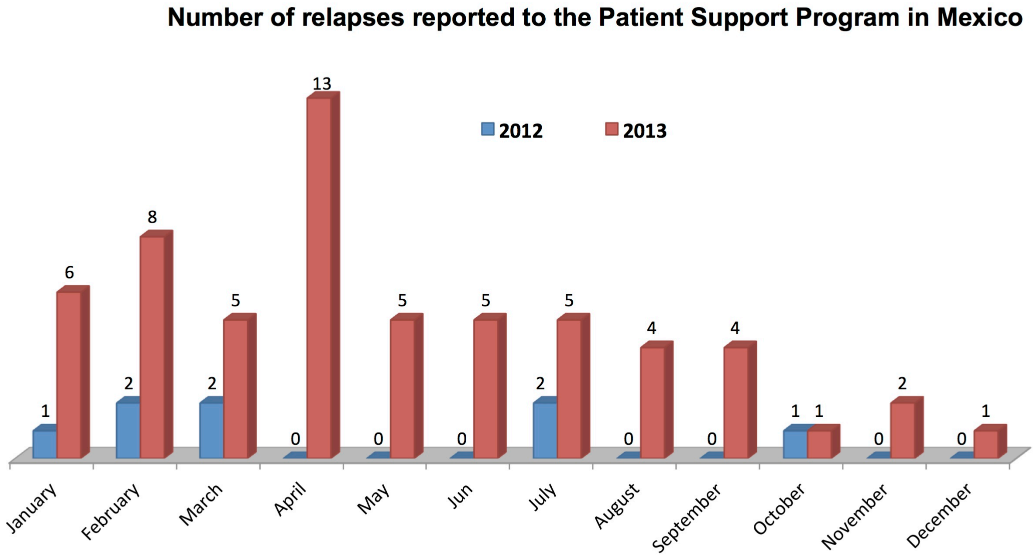

SUPPLEMENTARY FIGURE 3

a) First Principal Component accounts for experimental factor (Treatment Time (6,12, 24 hours))

b) PCA demonstrates batch effect correction required for scan date; legend as in (c)

c) After batch correction, PCA shows more uniform distribution of scan dates

a)

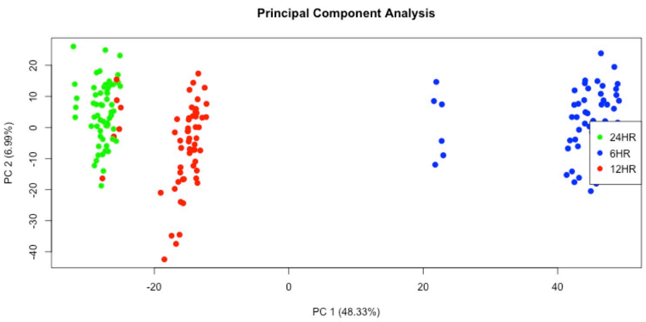

b)

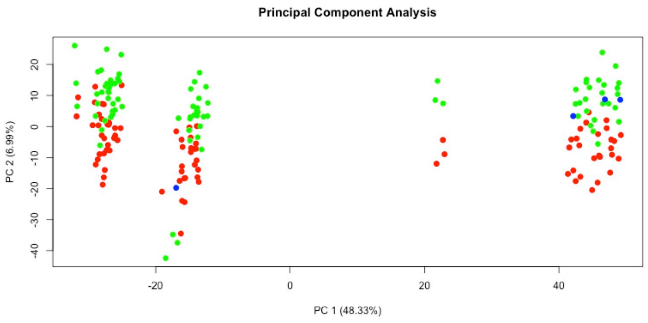

c)

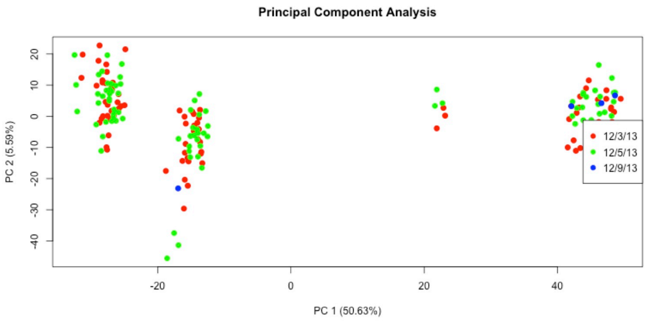

SUPPLEMENTARY FIGURE 4

Boxplots showing maximum and minimum expression values for Probioglat (red lines) and Copaxone (blue lines)

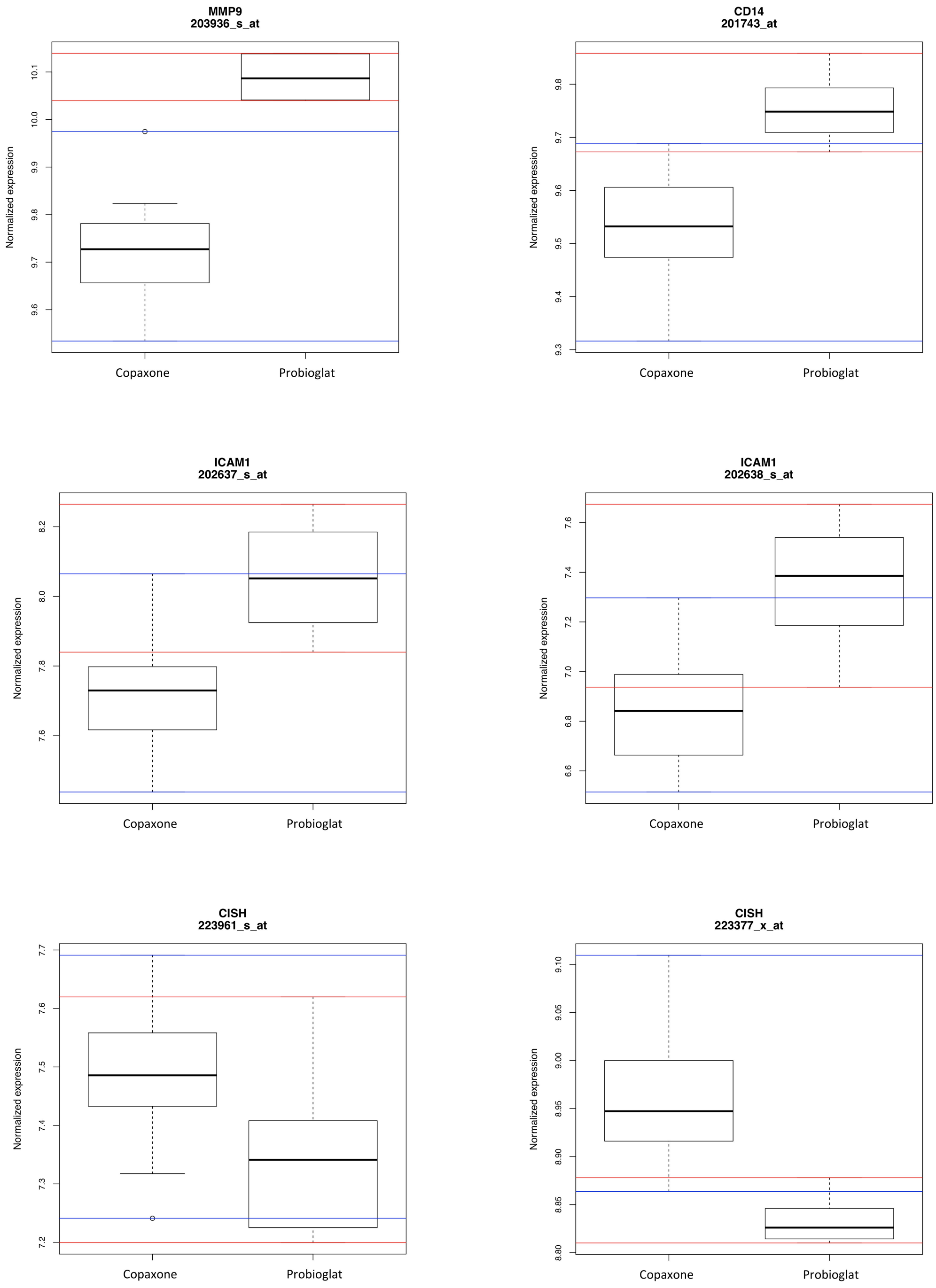

SUPPLEMENTARY FIGURE 5

CCL5, CXCL10 and MMP-9 protein levels are higher with Probioglat vs Copaxone at 24 hr, consistent with mRNA level observations by RT-PCR and microarray in THP-1 cells.

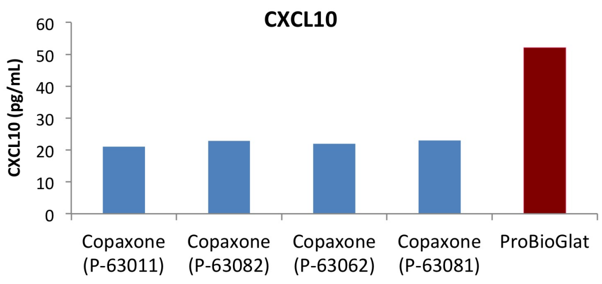

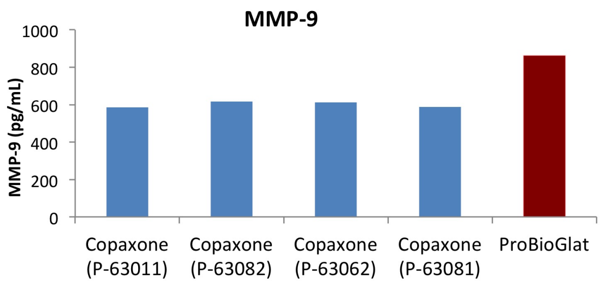

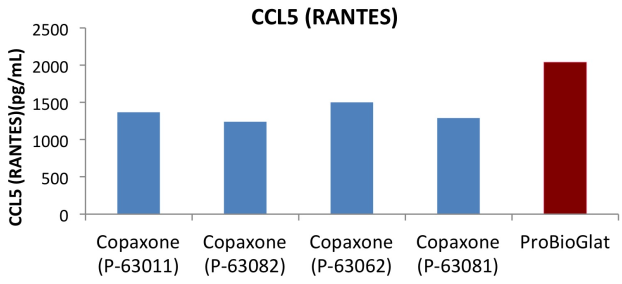

CCL2 and IL-10 protein levels are higher with Probioglat vs Copaxone at 24 hr, consistent with mRNA level observations by microarray (not tested by RT-PCR in THP-1 cells).

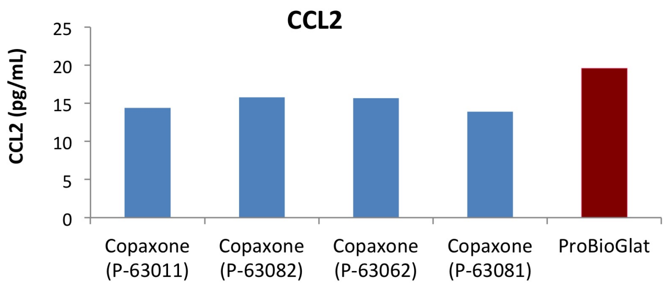

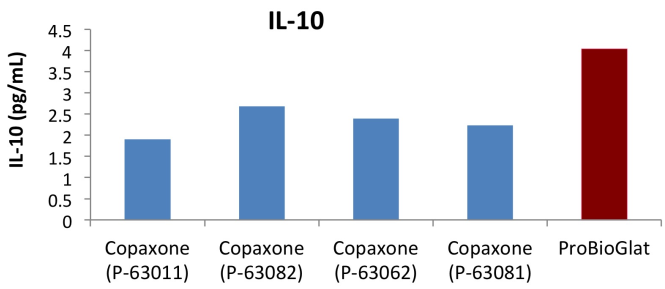

SUPPLEMENTARY TABLE 1

Probesets significantly modulated by GA relative to mannitol control at 6 hours:
(probesets subject to cutoffs of |FC|>1.5 and p-value<1e-5 are shown)

Upregulated:

ID	Gene	raw.FC	AveExpr	P.Value	adj.P.Val
201005_at	CD9	3.77232321	10.8126963	1.14E-34	6.23E-30
206835_at	STATH	7.52573433	10.4825707	1.00E-33	2.74E-29
1553982_a_at	RAB7B	2.26468475	11.7452289	1.26E-31	2.30E-27
242871_at	PAQR5	3.9374814	9.90015385	7.51E-31	1.01E-26
1555759_a_at	CCL5	2.21501539	13.5769189	9.21E-31	1.01E-26
228766_at	---	2.3291844	10.5559717	3.35E-30	3.06E-26
209555_s_at	CD36	2.44281389	9.32514677	7.64E-29	5.66E-25
203939_at	NT5E	5.3338766	8.32269063	8.29E-29	5.66E-25
208789_at	PTRF	2.82016222	9.15275301	1.88E-28	1.14E-24
230266_at	RAB7B	2.23824891	11.7520076	2.34E-28	1.28E-24
206488_s_at	CD36	2.15872949	10.8868374	3.67E-28	1.83E-24
208121_s_at	PTPRO	2.14699192	9.81795145	4.30E-28	1.96E-24
203037_s_at	MTSS1	3.1858517	11.4020576	7.89E-28	3.32E-24
1405_i_at	CCL5	2.29122708	13.3028093	2.79E-27	1.06E-23
204655_at	CCL5	2.25036905	13.1626051	2.90E-27	1.06E-23
206171_at	ADORA3	2.10036549	10.0753041	3.42E-27	1.17E-23
219386_s_at	SLAMF8	2.98872777	10.0302719	3.80E-27	1.22E-23
226218_at	---	3.50148157	7.21345525	1.83E-26	5.55E-23
218559_s_at	MAFB	2.64025573	12.4234341	3.40E-26	9.78E-23
223125_s_at	C1ORF21	2.21866076	11.0023707	4.15E-26	1.13E-22
203887_s_at	THBD	1.79337816	12.8191102	4.45E-26	1.15E-22
227265_at	KIAA1505	2.50321069	9.85031558	4.61E-26	1.15E-22
205495_s_at	GNLY	2.40116081	7.73128126	5.34E-26	1.27E-22
216250_s_at	LPXN	2.1444933	11.2374219	1.42E-25	3.23E-22
237252_at	THBD	2.06547107	9.51011605	2.22E-25	4.86E-22
235457_at	MAML2	2.62488399	8.91497905	5.79E-25	1.22E-21
225171_at	ARHGAP18	1.83225951	10.7080346	6.66E-25	1.35E-21
204684_at	NPTX1	2.87807656	10.9346668	1.40E-24	2.74E-21
230925_at	APBB1IP	1.94283823	12.7655513	2.02E-24	3.81E-21
201590_x_at	ANXA2	1.68959054	13.1436175	3.46E-24	6.31E-21
206157_at	PTX3	1.98670615	11.2102165	3.62E-24	6.38E-21
216080_s_at	FADS3	2.27239911	9.71739825	3.75E-24	6.39E-21
221463_at	CCL24	3.58807723	8.40481021	3.93E-24	6.39E-21
210427_x_at	ANXA2	1.67415609	13.1118777	3.98E-24	6.39E-21

213503_x_at	ANXA2	1.72399354	13.1135447	4.18E-24	6.51E-21
238681_at	GDPD1	2.92185146	7.19901556	4.29E-24	6.51E-21
202458_at	PRSS23	2.98100135	7.79000143	5.03E-24	7.43E-21
203761_at	SLA	1.78030673	11.2608124	6.52E-24	9.38E-21
1558397_at	---	2.20691751	9.17869031	8.35E-24	1.17E-20
214054_at	DOK2	1.67191836	12.2431311	8.80E-24	1.20E-20
228937_at	C13ORF31	2.42816683	9.90878354	9.66E-24	1.29E-20
211676_s_at	IFNGR1	1.64276331	12.0995889	1.08E-23	1.41E-20
230966_at	IL4I1	3.68319718	10.6065738	1.18E-23	1.50E-20
208816_x_at	ANXA2P2	1.61606927	11.6007732	1.33E-23	1.66E-20
204912_at	IL10RA	1.88097974	10.4596073	1.53E-23	1.83E-20
220014_at	LOC51334	3.95258058	6.49298277	1.54E-23	1.83E-20
218856_at	TNFRSF21	1.97277863	11.9973199	2.06E-23	2.40E-20
34210_at	CD52	1.74841624	11.3194919	2.57E-23	2.92E-20
211564_s_at	PDLIM4	2.13518707	9.68326105	2.72E-23	3.03E-20
223502_s_at	TNFSF13B	1.73530598	10.5512407	2.94E-23	3.21E-20
202727_s_at	IFNGR1	1.5463282	12.4669611	3.25E-23	3.48E-20
201243_s_at	ATP1B1	2.26738679	11.039008	4.33E-23	4.56E-20
209606_at	PSCDBP	2.53767762	8.05776375	4.93E-23	5.08E-20
222670_s_at	MAFB	2.65652588	11.7521547	6.27E-23	6.35E-20
223394_at	SERTAD1	1.65031524	9.76915308	6.91E-23	6.87E-20
214175_x_at	PDLIM4	1.85137891	9.80931564	8.66E-23	8.45E-20
223660_at	ADORA3	1.89720977	9.3830598	9.01E-23	8.64E-20
203217_s_at	ST3GAL5	1.78903169	9.10901818	1.17E-22	1.11E-19
204257_at	FADS3	1.78672893	9.74459459	1.23E-22	1.14E-19
223398_at	C9ORF89	1.54418891	11.0771528	1.25E-22	1.14E-19
211986_at	AHNAK	1.64967948	12.0209047	1.63E-22	1.45E-19
201278_at	DAB2	1.56823308	9.36397364	1.64E-22	1.45E-19
229797_at	MCOLN3	2.09980647	10.05118	2.01E-22	1.75E-19
202284_s_at	CDKN1A	1.789083	11.1500265	2.69E-22	2.22E-19
228579_at	---	1.73158064	8.85026747	3.40E-22	2.78E-19
204653_at	TFAP2A	1.98518712	8.32878609	5.70E-22	4.39E-19
201952_at	ALCAM	1.52577304	12.9059918	6.14E-22	4.66E-19
202087_s_at	CTSL	1.91284953	11.1924209	6.47E-22	4.84E-19
208978_at	CRIP2	1.99640335	10.1060568	6.82E-22	5.04E-19
219434_at	TREM1	1.77683606	11.1148543	6.95E-22	5.07E-19
235911_at	MFI2	1.81455526	10.1578351	7.75E-22	5.57E-19
225173_at	ARHGAP18	1.85956949	10.0159515	7.95E-22	5.65E-19
204661_at	CD52	1.6752998	11.8608631	9.08E-22	6.28E-19
202756_s_at	GPC1	1.88792383	8.93231998	9.08E-22	6.28E-19
227240_at	NGEF	1.97393626	9.35225393	9.96E-22	6.66E-19
220484_at	MCOLN3	2.10889009	9.93061659	9.98E-22	6.66E-19

214297_at	CSPG4	1.98668454	8.74794884	9.99E-22	6.66E-19
203355_s_at	PSD3	1.68707305	9.97354098	1.13E-21	7.47E-19
212386_at	---	1.80977269	8.4443852	1.22E-21	7.91E-19
201242_s_at	ATP1B1	2.36020561	10.833866	1.62E-21	1.03E-18
203760_s_at	SLA	1.76467647	10.2984296	1.71E-21	1.07E-18
204475_at	MMP1	4.89045646	6.14908101	1.97E-21	1.21E-18
228964_at	PRDM1	2.42316104	9.16116313	2.05E-21	1.24E-18
205566_at	ABHD2	1.61609464	9.56280738	2.12E-21	1.27E-18
224990_at	LOC201895	1.75486717	9.56572435	2.15E-21	1.28E-18
214830_at	SLC38A6	1.70065597	9.60777602	2.38E-21	1.38E-18
222876_s_at	CENTA2	1.62337809	9.52111995	3.45E-21	1.92E-18
218501_at	ARHGEF3	1.72054917	9.02047119	3.66E-21	2.02E-18
206134_at	ADAMDEC1	1.90734854	8.46538539	3.83E-21	2.10E-18
219385_at	SLAMF8	2.3601546	8.87175449	4.64E-21	2.49E-18
212298_at	NRP1	2.26706147	9.0154209	5.27E-21	2.77E-18
1556314_a_at	---	1.94563197	9.96244884	5.97E-21	3.05E-18
204834_at	FGL2	2.41220628	9.04212862	7.43E-21	3.63E-18
227052_at	---	1.7292497	9.65138189	8.49E-21	4.11E-18
223501_at	---	1.77755998	10.4775991	9.58E-21	4.59E-18
238669_at	PTGS1	1.79526928	9.00713066	1.52E-20	7.11E-18
203936_s_at	MMP9	1.83742157	9.54268719	2.01E-20	9.33E-18
205128_x_at	PTGS1	1.50682893	10.8982825	2.46E-20	1.12E-17
223019_at	C9ORF88	1.71440506	9.42451447	2.48E-20	1.12E-17
218223_s_at	PLEKHO1	1.76062083	9.13759715	2.55E-20	1.14E-17
226545_at	CD109	2.22747108	10.1210564	2.62E-20	1.17E-17
37145_at	GNLY	2.52474166	7.62618078	2.77E-20	1.21E-17
211661_x_at	PTAFR	1.65459655	9.40348625	3.20E-20	1.39E-17
223434_at	GBP3	1.79281477	8.94069901	5.65E-20	2.39E-17
221011_s_at	LBH /// LOC653	2.14546482	7.91354368	6.67E-20	2.79E-17
227478_at	LOC284262	2.55523487	8.16720299	7.57E-20	3.09E-17
205933_at	SETBP1	2.06852059	8.31470678	9.65E-20	3.88E-17
223126_s_at	C1ORF21	2.0098249	9.34926366	1.00E-19	3.99E-17
214255_at	ATP10A	1.87404352	8.48741153	1.17E-19	4.64E-17
203888_at	THBD	1.74251135	12.1003768	1.32E-19	5.18E-17
239761_at	GCNT1	1.60737016	9.89061934	1.78E-19	6.90E-17
205505_at	GCNT1	1.68341734	9.51667582	1.80E-19	6.93E-17
203665_at	HMOX1	2.77855009	12.0276078	1.83E-19	7.00E-17
228490_at	ABHD2	1.7612845	9.44137483	1.92E-19	7.27E-17
213428_s_at	COL6A1	1.6365876	9.11385826	2.21E-19	8.27E-17
202748_at	GBP2	2.05214857	8.5030424	2.71E-19	1.01E-16
63825_at	ABHD2	1.60237791	9.12592031	3.53E-19	1.28E-16
205542_at	STEAP1	1.7513289	7.91561507	4.15E-19	1.49E-16

215813_s_at	PTGS1	1.51668643	10.9189252	4.31E-19	1.53E-16
205891_at	ADORA2B	1.56662939	10.2505629	4.70E-19	1.66E-16
218613_at	LOC653754	1.55398123	9.29556045	4.95E-19	1.73E-16
205076_s_at	MTMR11	1.59326395	11.2341764	5.05E-19	1.76E-16
212086_x_at	LMNA	1.50357006	12.1481348	5.14E-19	1.78E-16
203104_at	CSF1R	1.64383545	11.9446038	5.33E-19	1.83E-16
228368_at	ARHGAP20	2.23117651	8.70947261	5.37E-19	1.84E-16
227889_at	AYTL1	1.59282395	10.8879284	5.43E-19	1.84E-16
203797_at	VSNL1	1.7623632	9.20419419	5.45E-19	1.84E-16
210145_at	PLA2G4A	1.69920287	9.86166848	5.50E-19	1.84E-16
220307_at	CD244	1.51030196	11.2887381	6.64E-19	2.20E-16
201280_s_at	DAB2	1.6207775	8.250618	7.03E-19	2.30E-16
205898_at	CX3CR1	1.5834422	12.3392251	7.53E-19	2.45E-16
212171_x_at	VEGF	1.67886192	11.082533	8.08E-19	2.61E-16
205419_at	EBI2	1.87963971	10.3117587	8.24E-19	2.64E-16
242794_at	MAML3	1.71070972	8.2000023	8.71E-19	2.76E-16
1553141_at	C13ORF31	2.23957531	8.21534882	9.28E-19	2.90E-16
1556034_s_at	MTMR11	1.54656669	10.734173	9.32E-19	2.90E-16
202436_s_at	CYP1B1	2.02651623	11.7446834	1.35E-18	4.08E-16
205718_at	ITGB7	1.61859456	10.0388412	1.64E-18	4.92E-16
222062_at	IL27RA	1.84145262	8.80213383	1.77E-18	5.26E-16
1565752_at	FGD2	2.03973925	8.7032554	1.80E-18	5.32E-16
203140_at	BCL6	1.6321005	9.84693758	1.88E-18	5.54E-16
209568_s_at	RGL1	1.79068455	9.03375054	2.04E-18	5.92E-16
212977_at	CXCR7	1.95857569	9.06724806	2.73E-18	7.74E-16
225809_at	DKFZP564O082	2.61498482	6.81686881	3.21E-18	8.90E-16
223723_at	MFI2	1.83918768	7.73004392	3.45E-18	9.49E-16
204141_at	TUBB2A	1.92837874	7.9645182	3.55E-18	9.72E-16
219358_s_at	CENTA2	1.61743685	10.3689956	3.67E-18	9.97E-16
218280_x_at	HIST2H2AA ///	1.92645343	11.1017962	3.91E-18	1.05E-15
200884_at	CKB	1.62119168	10.3585418	3.91E-18	1.05E-15
213265_at	PGA5 /// LOC6	1.54256466	9.03735387	4.18E-18	1.12E-15
228499_at	PFKFB4	1.54265427	8.78361163	4.58E-18	1.22E-15
214581_x_at	TNFRSF21	1.96726151	10.3862452	5.13E-18	1.35E-15
219256_s_at	SH3TC1	1.81211933	8.14371592	5.59E-18	1.46E-15
227134_at	SYTL1	1.63431503	10.2085878	5.74E-18	1.50E-15
210512_s_at	VEGF	1.61269337	12.1528461	5.88E-18	1.52E-15
220066_at	CARD15	1.63208119	8.81134661	6.12E-18	1.58E-15
207610_s_at	EMR2	1.96904599	7.45239164	6.31E-18	1.62E-15
225337_at	ABHD2	1.52226303	10.585684	6.47E-18	1.65E-15
1553906_s_at	FGD2	1.56817402	11.6738144	8.15E-18	2.05E-15
210757_x_at	DAB2	1.51596174	9.71603785	8.84E-18	2.22E-15

205306_x_at	KMO	1.52538689	9.01246744	1.07E-17	2.64E-15
203980_at	FABP4	2.44504844	7.91709543	1.14E-17	2.81E-15
205798_at	IL7R	2.15224639	6.90018766	1.20E-17	2.94E-15
235299_at	---	2.92455443	6.38887226	1.23E-17	2.99E-15
201422_at	IFI30	1.53617841	12.1952	1.33E-17	3.23E-15
201951_at	ALCAM	1.56151412	11.2835886	1.36E-17	3.29E-15
219412_at	RAB38	1.69022517	7.7984244	1.40E-17	3.36E-15
214290_s_at	HIST2H2AA ///	1.8793137	12.5116024	1.77E-17	4.19E-15
223092_at	ANKH	1.74621066	9.31182429	1.93E-17	4.53E-15
1552553_a_at	CARD12	1.8300836	8.04038883	1.98E-17	4.61E-15
1565754_x_at	FGD2	2.05302245	8.76658914	1.98E-17	4.61E-15
1562475_at	DKFZP686O132	1.84932663	8.38987825	2.25E-17	5.18E-15
202435_s_at	CYP1B1	1.96043252	11.8853309	2.26E-17	5.18E-15
222877_at	NRP2	2.43002847	6.1162318	2.37E-17	5.41E-15
201125_s_at	ITGB5	1.55447321	9.61610321	2.48E-17	5.62E-15
204222_s_at	GLIPR1	1.64989211	10.4691144	3.30E-17	7.31E-15
1553142_at	C13ORF31	2.72312753	7.05894587	3.42E-17	7.51E-15
201279_s_at	DAB2	1.51128632	9.36621678	3.53E-17	7.64E-15
203234_at	UPP1	1.74387737	9.56780561	3.97E-17	8.52E-15
226136_at	---	1.5023264	9.021618	4.37E-17	9.27E-15
206206_at	CD180	1.61188665	9.82309399	6.95E-17	1.43E-14
212387_at	---	1.62401877	7.80816226	1.05E-16	2.07E-14
204268_at	S100A2	1.79310512	8.64238178	1.22E-16	2.38E-14
209122_at	ADFP	1.61478632	13.4092966	1.23E-16	2.41E-14
219994_at	APBB1IP	1.87740546	9.6208094	1.42E-16	2.74E-14
210095_s_at	IGFBP3	2.98798358	8.63072598	1.43E-16	2.74E-14
227948_at	FGD4	1.62553487	7.73111329	1.54E-16	2.92E-14
243483_at	TRPM8	2.13837999	7.16652124	1.62E-16	3.04E-14
212062_at	ATP9A	2.12888023	7.50745486	1.77E-16	3.29E-14
218502_s_at	TRPS1	1.61387671	8.89822927	1.95E-16	3.60E-14
213891_s_at	---	1.67207935	8.30917084	2.03E-16	3.68E-14
200878_at	EPAS1	1.51714595	9.20362299	2.04E-16	3.69E-14
201565_s_at	ID2	1.52829092	12.7810873	2.26E-16	4.06E-14
212190_at	SERPINE2	1.92452066	9.90116936	2.57E-16	4.60E-14
224480_s_at	LPAAT-THETA	1.96245654	8.74709429	2.64E-16	4.67E-14
220333_at	PAQR5	3.58344803	7.84174393	2.70E-16	4.76E-14
218589_at	P2RY5	1.65149795	9.3651465	3.00E-16	5.23E-14
225166_at	ARHGAP18	1.75622288	7.33398136	3.29E-16	5.64E-14
226066_at	MITF	1.750697	7.87439597	3.35E-16	5.73E-14
221211_s_at	C21ORF7	2.08949444	5.978895	3.41E-16	5.80E-14
225842_at	PHLDA1	2.51724542	8.55236084	3.48E-16	5.89E-14
1554992_at	RASGRF1	1.56735654	6.94135257	3.77E-16	6.35E-14

225097_at	HIPK2	1.50753986	10.7130384	3.84E-16	6.45E-14
212464_s_at	FN1	1.70809888	9.26484812	4.53E-16	7.53E-14
240076_at	---	1.5987436	8.40204375	4.66E-16	7.73E-14
203798_s_at	VSNL1	1.91644457	7.87623269	4.84E-16	7.92E-14
204575_s_at	MMP19 /// LO(	1.83367508	7.70439226	4.88E-16	7.96E-14
204465_s_at	INA	1.60147629	8.20221976	5.17E-16	8.36E-14
201566_x_at	ID2 /// ID2B	1.57371123	10.7833858	5.52E-16	8.87E-14
230360_at	GLDN	1.98086959	6.74193069	6.80E-16	1.07E-13
222146_s_at	TCF4	1.54556332	7.51472351	8.25E-16	1.28E-13
224341_x_at	TLR4	1.7396424	8.97197657	8.61E-16	1.32E-13
219637_at	ARMC9	1.80286893	7.06720581	8.87E-16	1.35E-13
211138_s_at	KMO	1.54711526	8.79132849	9.12E-16	1.38E-13
202437_s_at	CYP1B1	1.80921395	12.1893518	9.54E-16	1.43E-13
235458_at	HAVCR2	2.48536941	7.7122454	9.75E-16	1.46E-13
203922_s_at	CYBB	1.58449815	10.3814577	1.07E-15	1.58E-13
212143_s_at	IGFBP3	2.76854721	7.92968375	1.26E-15	1.82E-13
227716_at	UBXD5	1.51942114	9.002862	1.28E-15	1.83E-13
214211_at	FTH1	1.55999567	12.4970466	1.33E-15	1.90E-13
229004_at	---	1.51039984	9.12368109	1.55E-15	2.18E-13
202609_at	EPS8	1.5924097	8.52061192	1.82E-15	2.52E-13
217757_at	A2M	1.67532065	8.4520092	1.98E-15	2.73E-13
1552798_a_at	TLR4	1.62695353	9.04302518	2.09E-15	2.88E-13
57715_at	FAM26B	1.79873285	7.75582627	2.40E-15	3.27E-13
226282_at	---	1.60485814	9.41013847	2.84E-15	3.80E-13
222838_at	SLAMF7	1.86121844	7.05728625	3.11E-15	4.13E-13
216442_x_at	FN1	1.69780521	10.1409361	3.73E-15	4.87E-13
201324_at	EMP1	2.82050293	6.11305975	3.81E-15	4.94E-13
210264_at	GPR35	1.59109619	9.60072153	3.91E-15	5.05E-13
222858_s_at	DAPP1	1.6313133	10.6750257	4.54E-15	5.78E-13
209684_at	RIN2	2.16288563	7.25953396	5.48E-15	6.84E-13
201069_at	MMP2	1.51386957	8.69855532	6.09E-15	7.55E-13
204998_s_at	ATF5	1.63500149	10.6267979	6.14E-15	7.60E-13
202434_s_at	CYP1B1	2.2027314	9.83119314	6.94E-15	8.52E-13
205099_s_at	CCR1	1.57264838	9.76018397	7.25E-15	8.85E-13
207233_s_at	MITF	1.66865012	8.30250431	8.03E-15	9.72E-13
205552_s_at	OAS1	1.79452532	7.29881386	8.53E-15	1.02E-12
226550_at	---	1.79237731	7.77105086	9.20E-15	1.09E-12
212096_s_at	MTUS1	1.62315248	8.50418445	9.61E-15	1.13E-12
222651_s_at	TRPS1	1.5940306	10.0878238	9.64E-15	1.13E-12
211719_x_at	FN1	1.74293625	10.1007312	9.80E-15	1.15E-12
227609_at	EPSTI1	1.73531372	7.62913716	1.06E-14	1.24E-12
1558105_a_at	---	1.89855059	6.81046182	1.07E-14	1.25E-12

225207_at	PDK4	2.24881528	8.76581849	1.08E-14	1.25E-12
237160_at	CCDC83	2.26816884	5.8347316	1.15E-14	1.33E-12
211026_s_at	MGLL	1.80987658	8.99536888	1.33E-14	1.53E-12
229450_at	---	2.07979683	6.90012377	1.42E-14	1.62E-12
215602_at	FGD2	2.01984347	7.85983935	1.54E-14	1.74E-12
228708_at	RAB27B	1.75996236	8.57078526	1.63E-14	1.83E-12
210258_at	RGS13	2.4181722	5.06675503	1.77E-14	1.97E-12
210895_s_at	CD86	1.74036933	7.80363779	1.88E-14	2.07E-12
223672_at	SGIP1	1.58033388	8.69396513	1.96E-14	2.14E-12
210513_s_at	VEGF	1.62960752	9.66705476	2.08E-14	2.25E-12
212614_at	ARID5B	1.64330835	6.34592171	2.09E-14	2.26E-12
1553995_a_at	NT5E	2.06345466	6.49987213	2.17E-14	2.32E-12
233540_s_at	CDK5RAP2	1.77928193	10.6899414	2.34E-14	2.48E-12
204059_s_at	ME1	1.78078662	11.5066468	2.46E-14	2.58E-12
211527_x_at	VEGF	1.68079685	9.90807927	2.48E-14	2.60E-12
214724_at	DIXDC1	1.55892061	7.67060007	2.63E-14	2.74E-12
227556_at	NME7	2.07486651	9.31851129	2.92E-14	3.02E-12
237442_at	APBB1IP	1.97657406	7.17373032	3.09E-14	3.18E-12
224218_s_at	TRPS1	1.52587932	8.16882094	3.38E-14	3.46E-12
225188_at	RAPH1	1.90298066	6.31647018	3.68E-14	3.73E-12
244414_at	MAML2	2.39160043	8.05798482	3.70E-14	3.74E-12
209906_at	C3AR1	1.62034968	8.32122922	3.73E-14	3.76E-12
210510_s_at	NRP1	1.56055098	7.3171434	3.77E-14	3.78E-12
226629_at	SLC43A2	1.57641868	8.50211433	3.97E-14	3.94E-12
237030_at	ACPP	1.53890938	8.05225501	4.15E-14	4.10E-12
213338_at	TMEM158	1.73773739	10.7585184	4.18E-14	4.13E-12
219926_at	POPDC3	2.12181972	6.63647483	5.99E-14	5.68E-12
200897_s_at	PALLD	1.64882729	8.35346573	6.07E-14	5.74E-12
208436_s_at	IRF7	1.55075885	8.74418094	6.34E-14	5.96E-12
228438_at	TRPA1	1.65933581	10.3950167	6.81E-14	6.30E-12
227747_at	---	1.55198677	7.93194342	6.84E-14	6.32E-12
1554867_a_at	LOC51334	1.86507425	7.40812044	6.90E-14	6.36E-12
1563445_x_at	CTSLL3	1.82851184	6.84405898	9.09E-14	8.12E-12
242903_at	IFNGR1	1.7678462	7.97757472	9.49E-14	8.42E-12
229900_at	CD109	1.54089288	8.62103824	1.05E-13	9.24E-12
210495_x_at	FN1	1.61505134	10.0973935	1.07E-13	9.43E-12
218854_at	SART2	1.54263807	8.36626559	1.13E-13	9.81E-12
224989_at	---	1.78010467	7.75893731	1.17E-13	1.02E-11
238638_at	SLC37A2	1.63842433	9.93438242	1.31E-13	1.13E-11
212382_at	---	1.60505834	7.06996616	1.35E-13	1.15E-11
209047_at	AQP1	2.44653569	7.84720827	1.38E-13	1.17E-11
236345_at	TBXAS1	1.66860916	8.46774141	1.40E-13	1.18E-11

228873_at	COL22A1	1.56093003	7.43196243	1.41E-13	1.19E-11
239519_at	NRP1	2.19110297	5.55763801	1.43E-13	1.20E-11
220935_s_at	CDK5RAP2	1.84541222	10.4100106	1.54E-13	1.28E-11
1560228_at	SNAI3	1.91948596	7.8146015	1.56E-13	1.30E-11
218934_s_at	HSPB7	1.65040526	8.10534113	2.40E-13	1.90E-11
210146_x_at	LILRB2	1.9982938	6.76423766	2.86E-13	2.22E-11
209348_s_at	MAF	1.56472875	8.72338323	3.17E-13	2.42E-11
1558569_at	LOC645238	1.75050375	7.56283286	3.25E-13	2.47E-11
243856_at	---	1.73115631	8.03640769	3.81E-13	2.86E-11
223798_at	SLC41A2	1.94681117	6.54582797	3.90E-13	2.92E-11
215990_s_at	BCL6	1.50671943	8.04953142	4.81E-13	3.52E-11
202869_at	OAS1	1.63942747	6.73520018	5.30E-13	3.86E-11
225631_at	KIAA1706	1.53753899	7.78142994	5.56E-13	4.03E-11
204116_at	IL2RG	1.53970115	7.99806857	5.88E-13	4.22E-11
225189_s_at	RAPH1	1.85367315	6.14549622	6.27E-13	4.47E-11
211066_x_at	PCDHGC3 /// P	1.58678656	10.4111947	7.90E-13	5.53E-11
221266_s_at	TM7SF4	1.83671444	6.22762311	8.02E-13	5.60E-11
1560485_at	HIVEP1	1.6480621	6.99003908	8.06E-13	5.62E-11
204881_s_at	UGCG	1.5102382	9.51481557	8.38E-13	5.83E-11
235286_at	CKLF	1.61741462	8.34288205	9.26E-13	6.36E-11
205003_at	DOCK4	1.5112689	8.20388936	1.02E-12	6.92E-11
218686_s_at	RHBDF1	1.51723286	7.92482272	1.05E-12	7.08E-11
221565_s_at	FAM26B	1.59642371	7.91366407	1.09E-12	7.32E-11
206675_s_at	SKIL	1.61042514	7.42083013	1.16E-12	7.69E-11
242358_at	---	2.20249712	6.04968315	1.24E-12	8.20E-11
241392_at	TMEM39A	1.61384348	7.05009494	1.87E-12	1.19E-10
232333_at	MAML2	1.68481156	8.01950459	2.06E-12	1.30E-10
228762_at	LFNG	1.55190457	9.0403074	2.08E-12	1.31E-10
242907_at	GBP2	2.14738965	6.03707956	2.72E-12	1.64E-10
203753_at	TCF4	1.50293289	7.66442858	2.78E-12	1.68E-10
207542_s_at	AQP1	1.69421791	6.82177348	3.02E-12	1.81E-10
242157_at	---	1.57383098	8.76647265	3.26E-12	1.94E-10
204058_at	ME1	1.73420796	9.51508263	3.53E-12	2.08E-10
209921_at	SLC7A11	1.94348494	9.29226579	3.63E-12	2.13E-10
211030_s_at	SLC6A6	1.51035569	7.57360907	3.78E-12	2.21E-10
209392_at	ENPP2	1.82310668	5.23034508	4.76E-12	2.73E-10
243894_at	SLC41A2	1.69547735	5.15085274	4.80E-12	2.75E-10
229937_x_at	LILRB1	1.58545389	7.41501062	5.00E-12	2.84E-10
214857_at	C10ORF95	1.60255368	7.13065345	5.02E-12	2.85E-10
230944_at	MGC45491	1.55203348	7.47887362	5.53E-12	3.14E-10
228057_at	DDIT4L	1.68290143	9.61033195	6.99E-12	3.82E-10
204105_s_at	NRCAM	2.27634648	6.95288228	7.49E-12	4.07E-10

205681_at	BCL2A1	1.64702983	9.50173198	8.19E-12	4.40E-10
242405_at	MAML2	1.86052011	7.15057318	1.10E-11	5.73E-10
217678_at	SLC7A11	1.75913555	8.8692241	1.22E-11	6.25E-10
211962_s_at	ZFP36L1	1.59912563	8.8232205	1.23E-11	6.27E-10
221060_s_at	TLR4	1.53812487	8.81340284	2.27E-11	1.08E-09
200907_s_at	PALLD	1.50739688	7.44132987	2.28E-11	1.08E-09
228918_at	SLC43A2	1.66364865	7.20166079	2.29E-11	1.09E-09
1555606_a_at	GDPD1	1.51121338	6.61034166	2.39E-11	1.13E-09
228450_at	PLEKHA7	1.50941089	6.45253703	2.70E-11	1.26E-09
229221_at	---	1.60263592	8.59003199	4.03E-11	1.79E-09
229635_at	LOC643424	1.53074512	7.55215178	4.38E-11	1.94E-09
1569149_at	PDLIM7	1.54849621	9.14969141	5.56E-11	2.41E-09
207433_at	IL10	1.52400243	5.63708634	7.49E-11	3.14E-09
221815_at	ABHD2	1.55904737	8.11542769	1.01E-10	4.08E-09
203060_s_at	PAPSS2	1.5764103	7.75963014	1.25E-10	4.94E-09
232746_at	CXCR7	2.0719893	6.54641126	1.43E-10	5.55E-09
1560960_at	MDGA1	1.97885133	5.71541524	1.47E-10	5.70E-09
234645_at	MAML2	1.87005824	6.53786268	1.72E-10	6.50E-09
223596_at	SLC12A6	1.50709112	7.32492551	1.75E-10	6.60E-09
87100_at	ABHD2	1.51939681	7.38326685	1.79E-10	6.75E-09
213931_at	ID2 /// ID2B	2.71874933	9.84931953	1.87E-10	7.02E-09
216874_at	DKFZP686O132	1.70886875	5.93467446	2.11E-10	7.76E-09
244375_at	EVL	1.56523772	7.93537524	2.24E-10	8.16E-09
1569150_x_at	PDLIM7	1.54117409	9.37943359	2.24E-10	8.18E-09
214453_s_at	IFI44	1.79674041	6.65852583	2.78E-10	9.87E-09
204999_s_at	ATF5	1.64452594	9.14215678	3.41E-10	1.18E-08
215836_s_at	PCDHGC3 /// P	1.52441384	9.29079122	4.72E-10	1.60E-08
208161_s_at	ABCC3	1.61726316	8.40189868	5.05E-10	1.69E-08
202827_s_at	MMP14	1.62314882	10.2030389	5.39E-10	1.78E-08
208712_at	CCND1	1.5393933	7.40438263	6.16E-10	2.01E-08
223939_at	SUCNR1	1.57537424	8.40597144	7.17E-10	2.32E-08
205960_at	PDK4	1.69465703	7.19094792	1.28E-09	3.84E-08
214841_at	CNIH3	1.56253317	6.14111085	1.40E-09	4.15E-08
209993_at	ABCB1	1.58407983	5.06880643	1.52E-09	4.48E-08
1557938_s_at	PTRF	1.50339198	6.8700736	1.62E-09	4.73E-08
232068_s_at	TLR4	1.74396268	6.76798691	1.84E-09	5.28E-08
242321_at	---	1.6662739	6.52856887	2.12E-09	5.99E-08
219574_at	MARCH1	1.64000788	5.55000344	3.54E-09	9.43E-08
202638_s_at	ICAM1	1.58753294	6.72394157	3.76E-09	9.97E-08
219496_at	ANKRD57	1.50970429	7.47583744	4.86E-09	1.26E-07
1555756_a_at	CLEC7A	1.65932872	7.0882743	5.25E-09	1.35E-07
241929_at	CD36	1.82152709	8.32014251	5.66E-09	1.44E-07

1556423_at	VASH1	1.55220936	7.02874938	8.01E-09	1.97E-07
229435_at	GLIS3	1.5824943	4.92660385	8.07E-09	1.98E-07
217997_at	PHLDA1	2.01871208	7.37991533	8.18E-09	2.00E-07
235944_at	HMCN1	1.51434592	7.331681	9.15E-09	2.21E-07
206637_at	P2RY14	1.69022854	4.58701985	9.57E-09	2.30E-07
213293_s_at	TRIM22	1.54165851	6.59931282	9.57E-09	2.30E-07
238581_at	GBP5	1.57383447	7.67594481	1.03E-08	2.46E-07
1558404_at	LOC644242 ///	1.85121181	5.31545142	1.46E-08	3.35E-07
230559_x_at	FGD4	1.51987215	5.45151267	1.69E-08	3.83E-07
1553151_at	ATP6V0D2	1.52950999	6.78084809	1.69E-08	3.84E-07
1554285_at	HAVCR2	1.64907555	7.49492704	3.00E-08	6.38E-07
244579_at	TRPS1	1.5354261	8.45494277	5.93E-08	1.16E-06
228640_at	---	1.5809974	4.58101232	7.52E-08	1.43E-06
210004_at	OLR1	1.59400178	5.26939608	8.40E-08	1.58E-06
243556_at	NGEF	1.50840974	6.73352627	1.18E-07	2.14E-06
210360_s_at	MTSS1	1.7452905	6.18923946	1.40E-07	2.49E-06
217999_s_at	PHLDA1	1.73310668	6.08679858	2.07E-07	3.52E-06
227062_at	TNCRNA	1.56840333	10.0441571	2.18E-07	3.69E-06
237904_at	ADORA3	1.52521897	6.15098521	2.49E-07	4.15E-06
1563621_at	KIAA1706	1.55572929	7.60284745	6.48E-07	9.74E-06

Downregulated:

ID	Gene	raw.FC	AveExpr	P.Value	adj.P.Val
207725_at	POU4F2	-2.8001105	9.19852692	2.12E-22	1.81E-19
212993_at	---	-1.8809512	9.86599984	2.25E-22	1.89E-19
211421_s_at	RET	-2.0125505	9.36968529	3.53E-22	2.84E-19
227899_at	VIT	-1.8754322	7.70929876	4.25E-22	3.36E-19
225224_at	C20ORF112	-2.04379	8.5964785	1.37E-21	8.79E-19
213361_at	TDRD7	-1.8488643	10.2732718	2.93E-21	1.67E-18
214539_at	SERPINB10	-2.1313083	8.01802953	3.16E-21	1.78E-18
235275_at	OXCT2	-2.0229254	12.4009174	4.06E-21	2.20E-18
203685_at	BCL2	-2.3112793	8.44431889	4.88E-21	2.59E-18
227037_at	LOC201164	-1.5468442	9.38990462	5.65E-21	2.94E-18
219714_s_at	CACNA2D3	-2.206206	9.82724428	5.72E-21	2.95E-18
206643_at	HAL	-1.9307917	9.28853464	6.18E-21	3.10E-18
211144_x_at	TRGC2 /// TRG\	-1.5748474	10.586818	7.12E-21	3.54E-18
217521_at	---	-1.7887534	8.38380263	7.38E-21	3.63E-18
208206_s_at	RASGRP2	-1.9706562	9.9264082	1.25E-20	5.95E-18
228977_at	IL17D	-2.1132305	7.51309566	2.19E-20	1.01E-17

209813_x_at	TRGC2 /// TRG\	-1.6036338	10.7697475	3.87E-20	1.65E-17
204614_at	SERPINB2	-2.3610663	10.8353035	6.10E-20	2.57E-17
212110_at	SLC39A14	-1.682778	10.5673949	7.23E-20	3.00E-17
202444_s_at	SPFH1	-1.8415073	9.34800962	7.37E-20	3.03E-17
216920_s_at	TRGC2 /// TRG\	-1.5704575	11.3491957	8.78E-20	3.56E-17
202441_at	SPFH1	-1.6532676	11.1411892	1.45E-19	5.66E-17
211919_s_at	CXCR4	-1.5387599	11.0738986	3.40E-19	1.25E-16
204610_s_at	CCDC85B	-1.729942	9.65009386	6.61E-19	2.20E-16
200999_s_at	CKAP4	-1.6192589	11.5934013	8.23E-19	2.64E-16
207865_s_at	BMP8B	-2.145585	9.11410521	9.38E-19	2.90E-16
217028_at	CXCR4	-1.5274428	11.5438714	1.14E-18	3.50E-16
201968_s_at	PGM1	-1.5051483	9.06546368	2.12E-18	6.10E-16
219463_at	C20ORF103	-1.5731957	10.0186106	2.55E-18	7.26E-16
212242_at	TUBA1	-1.5626324	11.2879097	2.82E-18	7.96E-16
213484_at	---	-1.5736546	8.53538561	3.16E-18	8.83E-16
209201_x_at	CXCR4	-1.5448129	11.1194624	3.16E-18	8.83E-16
218858_at	DEPDC6	-1.7866919	7.55045943	5.06E-18	1.34E-15
202236_s_at	SLC16A1	-1.5384699	10.1675739	9.26E-18	2.30E-15
208158_s_at	OSBPL1A	-1.6142678	8.3746552	1.71E-17	4.06E-15
202932_at	YES1	-1.6455703	8.57014581	2.47E-17	5.62E-15
243209_at	KCNQ4	-2.2463943	6.9665965	2.56E-17	5.79E-15
215806_x_at	TRGC2 /// TRG\	-1.5328764	11.0634752	3.51E-17	7.61E-15
204521_at	C12ORF24	-1.5676281	10.1844282	3.88E-17	8.36E-15
204301_at	KBTBD11	-1.5574513	10.253905	4.37E-17	9.27E-15
227920_at	KIAA1553	-1.6307063	8.01525239	5.17E-17	1.09E-14
218251_at	MID1IP1	-1.559714	9.78570246	5.29E-17	1.11E-14
212646_at	RFTN1	-1.658315	8.57843565	5.38E-17	1.12E-14
222799_at	HSPC049	-1.5489442	8.60085628	1.10E-16	2.17E-14
227103_s_at	MGC2408	-1.581709	8.7267591	1.30E-16	2.51E-14
206067_s_at	WT1	-1.511783	9.85618462	1.37E-16	2.66E-14
214369_s_at	RASGRP2	-1.5808582	9.41805033	1.69E-16	3.15E-14
206589_at	GFI1	-1.5996464	11.7281473	1.99E-16	3.65E-14
225619_at	SLAIN1	-2.2453889	7.41012008	2.01E-16	3.68E-14
212660_at	PHF15	-1.69531	8.00752875	2.63E-16	4.66E-14
218971_s_at	HSPC049	-1.5087203	9.12721477	3.08E-16	5.33E-14
201690_s_at	TPD52	-1.5492402	9.27038373	3.25E-16	5.59E-14
229638_at	IRX3	-1.6570492	11.8844594	3.38E-16	5.76E-14
223062_s_at	PSAT1	-1.556602	9.43823571	4.70E-16	7.77E-14
225510_at	OAF	-1.6632727	10.1142626	6.67E-16	1.05E-13
209485_s_at	OSBPL1A	-1.6049308	8.36910929	7.34E-16	1.15E-13
204432_at	SOX12	-1.7584476	6.5781916	8.71E-16	1.33E-13
205768_s_at	SLC27A2	-1.6303006	7.69181894	8.98E-16	1.36E-13

211576_s_at	SLC19A1	-1.5339487	9.8247482	9.81E-16	1.46E-13
201688_s_at	TPD52	-1.6048433	8.65393713	9.93E-16	1.48E-13
50314_i_at	C20ORF27	-1.561172	9.67877203	1.14E-15	1.66E-13
1553436_at	MUC19	-1.64037	7.99717726	1.18E-15	1.71E-13
202800_at	SLC1A3	-1.5176496	8.18097955	1.51E-15	2.13E-13
41037_at	TEAD4	-1.6405647	6.31029525	1.70E-15	2.36E-13
216953_s_at	WT1	-1.5276508	7.76890727	2.15E-15	2.95E-13
228055_at	NAPSB	-1.5385754	10.1648177	2.61E-15	3.52E-13
218424_s_at	STEAP3	-1.7692882	7.88028032	2.78E-15	3.74E-13
212855_at	DCUN1D4	-1.508562	7.88887861	2.92E-15	3.90E-13
202933_s_at	YES1	-1.5437793	9.12374841	3.76E-15	4.89E-13
1553138_a_at	ANKRD41	-2.0146608	6.96935627	6.26E-15	7.73E-13
227242_s_at	EBF3	-1.8363477	4.86769268	8.15E-15	9.84E-13
237265_at	C16ORF73	-1.5296704	9.74165113	9.60E-15	1.13E-12
213478_at	KIAA1026	-1.8330762	7.39399329	1.16E-14	1.34E-12
225081_s_at	CDCA7L	-1.5491727	10.2951966	1.46E-14	1.66E-12
211299_s_at	FLOT2	-1.6145204	9.10864173	1.58E-14	1.79E-12
213568_at	OSR2	-2.2462672	5.99962469	1.64E-14	1.83E-12
227662_at	SYNPO2	-1.5973696	8.62811124	1.76E-14	1.96E-12
200998_s_at	CKAP4	-1.6910951	9.77491302	2.08E-14	2.25E-12
209900_s_at	SLC16A1	-1.5885969	7.84437981	2.30E-14	2.43E-12
229084_at	CNTN4	-1.6415136	7.72735571	2.30E-14	2.43E-12
238694_at	---	-1.5762461	7.26420148	3.38E-14	3.46E-12
1555788_a_at	TRIB3	-1.5022329	9.92495817	3.64E-14	3.70E-12
221900_at	COL8A2	-1.5627579	10.2358313	4.09E-14	4.05E-12
225016_at	APCDD1	-1.6872345	6.47036912	4.21E-14	4.14E-12
225768_at	NR1D2	-1.7351352	6.8044854	5.44E-14	5.24E-12
222095_s_at	C1ORF76	-1.7301956	7.38672115	6.40E-14	6.00E-12
229103_at	WNT3	-1.7749895	5.55503782	7.97E-14	7.21E-12
205769_at	SLC27A2	-1.7824201	7.67753108	1.53E-13	1.27E-11
201689_s_at	TPD52	-1.645479	8.12663653	1.59E-13	1.31E-11
201350_at	FLOT2	-1.5079906	11.0344892	2.08E-13	1.68E-11
221648_s_at	C1ORF121	-1.517313	8.36815023	2.55E-13	2.00E-11
208078_s_at	SNF1LK	-1.597736	7.64505173	2.81E-13	2.18E-11
1562484_at	FLJ35848	-1.9050209	6.65405977	3.15E-13	2.41E-11
1555370_a_at	CAMTA1	-1.7040191	6.36041757	3.26E-13	2.47E-11
218376_s_at	MICAL1	-1.5055354	11.194367	3.44E-13	2.61E-11
204567_s_at	ABCG1	-1.8330717	5.8226155	3.51E-13	2.66E-11
39966_at	CSPG5	-1.6105172	7.68266153	4.12E-13	3.07E-11
218326_s_at	LGR4	-1.696564	6.525917	5.28E-13	3.85E-11
212430_at	RBM38	-1.5884137	8.88476901	5.78E-13	4.17E-11
227236_at	TSPAN2	-1.7695047	6.98434119	7.37E-13	5.19E-11

204794_at	DUSP2	-1.5957268	8.45706393	7.96E-13	5.57E-11
230888_at	HSPC049	-1.577802	7.20584208	9.14E-13	6.29E-11
212097_at	CAV1	-1.6628831	7.59130877	9.63E-13	6.57E-11
209353_s_at	C1ORF76	-1.7131519	7.30778535	1.02E-12	6.92E-11
201801_s_at	SLC29A1	-1.6246978	8.73200853	1.05E-12	7.08E-11
210279_at	GPR18	-1.5350315	8.98079125	1.29E-12	8.49E-11
52651_at	COL8A2	-1.577706	9.9516637	1.31E-12	8.57E-11
235758_at	PNMA6A	-1.5784572	7.86091427	1.34E-12	8.77E-11
222162_s_at	ADAMTS1	-1.842254	6.45805339	1.37E-12	8.95E-11
209369_at	ANXA3	-1.7449323	5.34631362	1.41E-12	9.18E-11
219911_s_at	SLCO4A1	-1.5143987	8.17455361	1.50E-12	9.71E-11
1557919_a_at	LOC648232	-1.5028156	12.5466889	1.73E-12	1.11E-10
219497_s_at	BCL11A	-1.5825853	9.14635109	1.85E-12	1.18E-10
232271_at	HNF4G	-1.5978388	7.3715225	2.04E-12	1.29E-10
206653_at	---	-1.5611734	6.70426513	2.13E-12	1.33E-10
225782_at	MSRB3	-1.5020089	8.17316381	2.54E-12	1.55E-10
223704_s_at	DMRT2	-1.6370445	6.61601092	5.67E-12	3.20E-10
1558613_at	OAF	-1.5776535	7.5183993	6.15E-12	3.44E-10
1555434_a_at	SLC39A14	-1.5483414	7.91388969	6.58E-12	3.65E-10
1556194_a_at	---	-1.7264827	7.80933003	1.10E-11	5.73E-10
219304_s_at	PDGFD	-1.5313053	8.14849618	1.10E-11	5.73E-10
200894_s_at	FKBP4	-1.6280907	8.06454238	1.28E-11	6.49E-10
210347_s_at	BCL11A	-1.5419287	7.82101671	1.36E-11	6.85E-10
221249_s_at	FAM117A	-1.5920148	8.42643515	1.45E-11	7.21E-10
239410_at	---	-1.521547	6.86497836	1.72E-11	8.46E-10
206039_at	RAB33A	-1.5487461	8.85562976	2.20E-11	1.05E-09
1554660_a_at	C1ORF71	-1.5203911	7.61313106	2.54E-11	1.19E-09
209776_s_at	SLC19A1	-1.5451778	7.72495148	2.90E-11	1.34E-09
1560495_at	---	-1.6478409	7.28259363	3.01E-11	1.38E-09
202234_s_at	SLC16A1	-1.5891823	7.76240785	4.97E-11	2.16E-09
207768_at	EGR4	-2.266679	5.08097601	7.18E-11	3.02E-09
207717_s_at	PKP2	-1.5155863	4.5970225	1.26E-10	4.97E-09
213912_at	KIAA0984	-1.5617265	6.49154577	1.30E-10	5.13E-09
222891_s_at	BCL11A	-1.5929549	8.42933212	2.00E-10	7.44E-09
213268_at	CAMTA1	-1.7460973	5.75010725	2.03E-10	7.52E-09
232007_at	---	-1.5199738	9.70608088	2.60E-10	9.33E-09
227099_s_at	LOC387763	-1.5087957	6.4279952	2.97E-10	1.05E-08
232614_at	BCL2	-1.6221488	7.30276639	2.99E-10	1.05E-08
222668_at	KCTD15	-1.5400712	5.79509816	3.31E-10	1.15E-08
213610_s_at	KLHL23	-1.5730988	7.2326013	4.60E-10	1.56E-08
206935_at	PCDH8	-1.6992238	5.66380311	6.65E-10	2.16E-08
203708_at	PDE4B	-1.5144333	7.44164625	8.10E-10	2.59E-08

1554298_a_at	WDR49	-1.5642625	6.77053726	8.63E-10	2.73E-08
212558_at	SPRY1	-1.6087624	5.95823849	1.41E-09	4.18E-08
242509_at	C16ORF74	-1.5318238	6.48465026	2.21E-09	6.20E-08
201939_at	PLK2	-1.5317787	7.43694803	5.43E-09	1.39E-07
242245_at	SYDE2	-1.5851454	4.93022084	5.57E-09	1.42E-07
204557_s_at	DZIP1	-1.5326546	5.01217049	5.85E-09	1.48E-07
204875_s_at	GMDS	-1.7607496	6.33236847	9.81E-09	2.35E-07
204285_s_at	PMAIP1	-1.7424137	10.9563243	1.15E-08	2.73E-07
213006_at	CEBPD	-1.9902998	8.08295825	1.47E-08	3.38E-07
1554830_a_at	STEAP3	-1.6156641	5.65856155	2.19E-08	4.84E-07
204286_s_at	PMAIP1	-1.7353429	9.45448346	6.05E-08	1.18E-06
228325_at	KIAA0146	-1.8819676	8.36526925	8.31E-08	1.57E-06
1569377_at	TMEM67	-1.9524092	4.4888277	1.11E-07	2.04E-06
242892_at	PER2	-2.1304461	6.97699912	1.72E-07	3.00E-06
1555847_a_at	LOC284454	-2.1798122	9.88866319	1.88E-07	3.24E-06

SUPPLEMENTARY TABLE 2

Pathways significantly enriched among top genes modulated by GA relative to mannitol control at 6 hours:
Top genes are defined by cutoffs of |FC|>1.5 and p-value<1e-5 to obtain lists of appropriate size for use with DAVID.

Enriched among top upregulated genes:

Term	Pvalue	Fold Enrichm	Benjamini
GO:0005886~plasma membrane	1.02E-19	2.41979941	2.18E-17
GO:0031226~intrinsic to plasma membrane	1.09E-11	3.26755853	7.78E-10
GO:0016020~membrane	8.59E-12	1.53815131	9.23E-10
GO:0005887~integral to plasma membrane	2.42E-11	3.24970302	1.04E-09
GO:0044459~plasma membrane part	1.97E-11	2.40372122	1.06E-09
GO:0005576~extracellular region	1.37E-10	2.82387307	4.90E-09
GO:0031224~intrinsic to membrane	4.97E-10	1.68368379	1.53E-08
GO:0004871~signal transducer activity	1.04E-10	2.53722457	2.51E-08
GO:0060089~molecular transducer activity	1.04E-10	2.53722457	2.51E-08
GO:0004872~receptor activity	6.20E-11	2.97630615	2.99E-08
GO:0044425~membrane part	1.40E-09	1.5389803	3.75E-08
GO:0016021~integral to membrane	1.98E-09	1.67551367	4.73E-08
GO:0044421~extracellular region part	3.24E-09	3.6100505	6.97E-08
GO:0004888~transmembrane receptor activity	2.35E-09	3.76803098	3.78E-07
GO:0006955~immune response	9.81E-10	3.57048831	6.34E-07
GO:0009611~response to wounding	3.29E-10	4.09715057	6.39E-07
GO:0009605~response to external stimulus	7.26E-10	3.12728281	7.04E-07
GO:0002376~immune system process	1.92E-09	2.85245261	9.30E-07
GO:0050896~response to stimulus	2.41E-09	1.85219081	9.36E-07
GO:0032501~multicellular organismal process	7.40E-09	1.76972744	2.39E-06
GO:0050793~regulation of developmental process	3.28E-08	3.16072861	9.08E-06
GO:0009897~external side of plasma membrane	5.99E-07	6.49667519	1.17E-05
GO:0051239~regulation of multicellular organismal process	7.17E-08	2.79159385	1.54E-05
GO:0006952~defense response	6.90E-08	3.41796458	1.67E-05
GO:0006954~inflammatory response	8.72E-08	4.47517285	1.69E-05
GO:0031012~extracellular matrix	1.51E-06	5.01711743	2.70E-05

GO:0048583~regulation of response to stimulus	1.68E-07	3.61268499	2.96E-05
GO:0005578~proteinaceous extracellular matrix	2.00E-06	5.30978261	3.07E-05
GO:0009986~cell surface	1.88E-06	4.06706753	3.10E-05
GO:0002682~regulation of immune system process	3.21E-07	3.76321353	5.19E-05
GO:0005615~extracellular space	6.96E-06	3.37129055	9.98E-05
GO:0048731~system development	7.36E-07	1.9176759	0.00010967
GO:0051707~response to other organism	1.25E-06	4.4259178	0.00017294
GO:0007275~multicellular organismal development	2.71E-06	1.74240626	0.00032825
GO:0032101~regulation of response to external stimulus	2.59E-06	6.28790109	0.00033427
GO:0022610~biological adhesion	3.65E-06	2.94001057	0.00039282
GO:0007155~cell adhesion	3.47E-06	2.94838667	0.00039594
GO:0048856~anatomical structure development	4.11E-06	1.79200644	0.00041952
GO:0065008~regulation of biological quality	5.75E-06	2.06736353	0.00050661
GO:0007166~cell surface receptor linked signal transduction	5.31E-06	2.24578872	0.00051425
GO:0050727~regulation of inflammatory response	5.72E-06	8.87043189	0.00052809
GO:0006950~response to stress	6.47E-06	1.89682191	0.0005449
GO:0032502~developmental process	9.45E-06	1.64267257	0.00073237
GO:0031347~regulation of defense response	9.45E-06	5.51937984	0.0007628
GO:0009607~response to biotic stimulus	1.20E-05	3.37558639	0.00089042
GO:0019955~cytokine binding	1.09E-05	6.97096043	0.00131926
GO:0050776~regulation of immune response	2.06E-05	4.29285099	0.00147588
GO:0040011~locomotion	2.38E-05	3.21025154	0.00164377
GO:0080134~regulation of response to stress	2.54E-05	3.70014291	0.00169407
GO:0007626~locomotory behavior	3.53E-05	4.08123158	0.00227722
GO:0002684~positive regulation of immune system process	4.09E-05	4.0245478	0.00255401
GO:0000267~cell fraction	0.00027348	1.99311488	0.00366864
GO:0045028~purinergic nucleotide receptor activity, G-protein coupled	5.12E-05	13.8685423	0.00411604
GO:0001608~nucleotide receptor activity, G-protein coupled	5.12E-05	13.8685423	0.00411604
GO:0004930~G-protein coupled receptor activity	4.66E-05	4.29264405	0.00449193
GO:0016502~nucleotide receptor activity	8.64E-05	12.5477288	0.00594437

GO:0001614~purinergic nucleotide receptor activity	8.64E-05	12.5477288	0.00594437
GO:0005626~insoluble fraction	0.00050614	2.11983643	0.00638231
GO:0048513~organ development	0.00010948	1.8456525	0.00640896
GO:0002697~regulation of immune effector process	0.00010851	6.00900225	0.00655072
GO:0051384~response to glucocorticoid stimulus	0.00011695	7.04601682	0.00664447
GO:0005624~membrane fraction	0.00064098	2.12816938	0.00762936
hsa04060:Cytokine-cytokine receptor interaction	7.88E-05	3.91086691	0.00816277
GO:0031349~positive regulation of defense response	0.00015337	6.7584243	0.00822279
GO:0048584~positive regulation of response to stimulus	0.00015308	3.78971503	0.00844122
GO:0031960~response to corticosteroid stimulus	0.00017474	6.62325581	0.00911158
GO:0042221~response to chemical stimulus	0.00023157	1.87929805	0.01115792
GO:0065007~biological regulation	0.00022752	1.23581669	0.01124356
GO:0050865~regulation of cell activation	0.00022283	4.29574375	0.01130118
GO:0048545~response to steroid hormone stimulus	0.00025981	4.21619294	0.0122074
GO:0006935~chemotaxis	0.00027256	4.65116279	0.01249951
GO:0042330~taxis	0.00027256	4.65116279	0.01249951
GO:0002703~regulation of leukocyte mediated immunity	0.0003028	7.42993441	0.01355657
GO:0002822~regulation of adaptive immune response based on somatic recombination of immune receptors built from immunoglobulin superfamily domains	0.00034918	7.24418605	0.01492816
GO:0001568~blood vessel development	0.00034714	3.47186797	0.01517609
GO:0030154~cell differentiation	0.00037713	1.79379845	0.01576601
GO:0001944~vasculature development	0.00039042	3.42764035	0.01597268
GO:0002819~regulation of adaptive immune response	0.00040094	7.06749858	0.0160609
GO:0051241~negative regulation of multicellular organismal process	0.0004538	4.90208078	0.01743952
GO:0051704~multi-organism process	0.00044925	2.22256907	0.01761538
GO:0007165~signal transduction	0.00048801	1.56692649	0.01837798
GO:0009617~response to bacterium	0.00051926	4.26756174	0.01917123
GO:0048519~negative regulation of biological process	0.00057125	1.62866946	0.01993331

GO:0050864~regulation of B cell activation	0.00056871	8.56455493	0.0202092
GO:0001525~angiogenesis	0.00056006	4.22401519	0.02027648
GO:0048869~cellular developmental process	0.00059519	1.73565404	0.02039329
GO:0002694~regulation of leukocyte activation	0.00064956	4.13953488	0.02184996
GO:0044243~multicellular organismal catabolic process	0.00070212	20.6976744	0.02319544
GO:0030574~collagen catabolic process	0.00070212	20.6976744	0.02319544
GO:0016477~cell migration	0.00071879	3.20321152	0.02334233
GO:0045595~regulation of cell differentiation	0.0007682	2.51728473	0.02373537
GO:0032879~regulation of localization	0.0007822	2.35870934	0.02378398
GO:0050729~positive regulation of inflammatory response	0.00076295	11.498708	0.0239569
GO:0007186~G-protein coupled receptor protein signaling pathway	0.00075328	2.72561968	0.0240464
GO:0051270~regulation of cell motion	0.00083518	3.6427907	0.02498336
GO:0048518~positive regulation of biological process	0.0009722	1.55920442	0.02858412
GO:0001816~cytokine production	0.00105123	7.52642706	0.0299652
GO:0007610~behavior	0.00104771	2.75968992	0.03031177
GO:0050878~regulation of body fluid levels	0.00117703	4.87004104	0.03300809
GO:0032496~response to lipopolysaccharide	0.0013154	5.68171455	0.03578575
GO:0002683~negative regulation of immune system process	0.0013154	5.68171455	0.03578575
GO:0051674~localization of cell	0.00131424	2.98966408	0.03626342
GO:0048870~cell motility	0.00131424	2.98966408	0.03626342
GO:0046903~secretion	0.00139387	3.16397571	0.03684834
GO:0006928~cell motion	0.0013811	2.46919625	0.03702169
GO:0048514~blood vessel morphogenesis	0.00142405	3.39812565	0.03712585
GO:0001501~skeletal system development	0.00146721	3.14395054	0.03772332
GO:0050867~positive regulation of cell activation	0.00151961	4.66426466	0.03853451
GO:0030247~polysaccharide binding	0.0007648	5.24382695	0.04514181
GO:0001871~pattern binding	0.0007648	5.24382695	0.04514181
GO:0004222~metalloendopeptidase activity	0.00087418	6.1483871	0.04585047
GO:0002698~negative regulation of immune effector process	0.00196103	15.0528541	0.0488232
GO:0032103~positive regulation of response to external stimulus	0.00201705	6.53610771	0.04892898
GO:0010033~response to organic substance	0.00199539	2.03004909	0.04902907

Enriched among top downregulated genes:

Term	Pvalue	Fold Enrichm	Benjamini
GO:0007275~multicellular organismal development	6.94E-06	2.16836735	0.00270611
GO:0048731~system development	5.04E-06	2.39616613	0.00294797
GO:0048513~organ development	2.61E-06	2.76008493	0.00305609
GO:0048856~anatomical structure development	3.69E-05	2.16450216	0.00861537
GO:0030154~cell differentiation	4.49E-05	2.55555556	0.00871888
GO:0032502~developmental process	3.28E-05	1.98412698	0.00955745
GO:0003006~reproductive developmental process	8.34E-05	6.25	0.01385786
GO:0048869~cellular developmental process	0.00010797	2.41090147	0.0156803
GO:0009653~anatomical structure morphogenesis	0.00030745	2.64317181	0.03921862

SUPPLEMENTARY TABLE 3

Table 3 shows modulated genes (note that FC<0 for GA-Probioglat comparison means upregulated in Probioglat-GA comparison) without presence/absence filtering at each timepoint.

Comparing GA to Probioglat (mannitol-corrected):

Upregulated:

6 hr:

Gene	ID	raw.FC	AveExpr	t	P.Value	adj.P.Val	B
MMP9	203936_s_at	-1.2926295	9.63366142	-9.0041791	5.02E-11	2.74E-06	13.793663
CXCL10	204533_at	-1.4567769	5.93611913	-6.8101441	4.11E-08	0.00056208	8.11662055
PRDM1	228964_at	-1.3131543	9.26923221	-6.9712916	2.48E-08	0.00056208	8.55501455
LPXN	216250_s_at	-1.1874257	11.315416	-6.8905168	3.19E-08	0.00056208	8.33560319
FABP4	203980_at	-1.3897081	8.03922119	-6.4609935	1.24E-07	0.00100306	7.15838496
---	240076_at	-1.2154985	8.47153306	-6.4502687	1.28E-07	0.00100306	7.12879005
COL6A1	213428_s_at	-1.1799797	9.17734162	-6.5227006	1.02E-07	0.00100306	7.3284926
SLC39A8	209267_s_at	-1.1409956	10.4538343	-6.3855772	1.58E-07	0.00107772	6.95009387
MGC5618	221477_s_at	-1.1617675	10.5471179	-6.2424898	2.48E-07	0.00135843	6.55382496
SLC39A8	219869_s_at	-1.14866	10.1877186	-6.2694824	2.28E-07	0.00135843	6.62867956
---	226218_at	-1.2937342	7.33564432	-6.1059571	3.84E-07	0.00140732	6.17456221
STEAP1	205542_at	-1.198521	7.98610593	-6.0852841	4.10E-07	0.00140732	6.11705174
CHST11	219634_at	-1.1616563	9.51077621	-6.0764318	4.21E-07	0.00140732	6.09241933
CD9	201005_at	-1.1480015	10.9097322	-6.0774378	4.20E-07	0.00140732	6.09521866
TNFSF13B	223502_s_at	-1.137131	10.6086468	-6.1814333	3.02E-07	0.00140732	6.38434731
LACTB	226354_at	-1.1146026	10.6969136	-6.1724213	3.10E-07	0.00140732	6.35931437
ARL6IP5	200761_s_at	-1.1108957	11.9357889	-6.0645684	4.38E-07	0.00140732	6.05940234
NFKBIE	203927_at	-1.1646409	10.0523732	-5.9997498	5.38E-07	0.00157615	5.87889643
SLIC1	228869_at	-1.1244476	10.0277009	-5.994027	5.48E-07	0.00157615	5.86295139
MMP1	204475_at	-1.5039262	6.32353561	-5.9312361	6.69E-07	0.00174156	5.68791885
SOD2	215223_s_at	-1.2035944	9.66361398	-5.9462273	6.38E-07	0.00174156	5.72972041
NFKBIA	201502_s_at	-1.1701699	11.8415908	-5.9153553	7.04E-07	0.0017486	5.64362855
ADAMDEC1	206134_at	-1.1861199	8.53747931	-5.7927992	1.04E-06	0.00247065	5.30158178
MPEG1	226841_at	-1.141713	9.90354597	-5.7187643	1.32E-06	0.00281664	5.09479213
ANXA2P2	208816_x_at	-1.1086074	11.6486476	-5.7130844	1.34E-06	0.00281664	5.07892372
---	244434_at	-1.2846909	6.48107186	-5.6742369	1.52E-06	0.00284109	4.97038261
CCL2	216598_s_at	-1.2516796	11.2421198	-5.6653825	1.56E-06	0.00284109	4.94564092
**ISG20**	204698_at	-1.2176821	6.18754674	-5.677111	1.50E-06	0.00284109	4.97841335
MTSS1	203037_s_at	-1.2132697	11.5042636	-5.6909681	1.44E-06	0.00284109	5.0171321
IFNGR1	211676_s_at	-1.1076638	12.1480464	-5.6472459	1.65E-06	0.0029127	4.89496045
---	230795_at	-1.1702144	9.26991641	-5.6333398	1.73E-06	0.00294926	4.85610012
NFE2L3	236471_at	-1.3257415	5.26403664	-5.6026446	1.90E-06	0.00315306	4.77032024
CD36	209555_s_at	-1.1414722	9.39991418	-5.5482888	2.26E-06	0.00363742	4.61841752
CYLD	221903_s_at	-1.1207309	9.54523467	-5.5045621	2.60E-06	0.00406002	4.49622693
ICAM1	202638_s_at	-1.4084929	6.8285265	-5.4886851	2.73E-06	0.00415135	4.45186392
KIAA1505	227265_at	-1.1673293	9.93164349	-5.4623712	2.97E-06	0.00423964	4.37834442
BID	227143_s_at	-1.1112086	11.5740779	-5.471422	2.89E-06	0.00423964	4.40363069
BID	211725_s_at	-1.1017724	11.8106494	-5.4568423	3.02E-06	0.00423964	4.36289801
IL10	207433_at	-1.2783883	5.71640286	-5.3801791	3.86E-06	0.00508408	4.14877517
ANXA2	201590_x_at	-1.1067663	13.1932317	-5.3762172	3.91E-06	0.00508408	4.13771284
TNFAIP6	206026_s_at	-1.2533604	5.46505459	-5.3471287	4.28E-06	0.00544542	4.0565035
CD14	201743_at	-1.1688628	9.56313815	-5.3162757	4.72E-06	0.00586803	3.97039297
ICAM1	202637_s_at	-1.253703	7.7078627	-5.2602772	5.64E-06	0.00674579	3.81417837
ANXA2	210427_x_at	-1.1021711	13.1600502	-5.2581893	5.68E-06	0.00674579	3.80835622
SYNJ2	212828_at	-1.1079961	9.8266126	-5.2259269	6.29E-06	0.00731141	3.71841109

GLIPR1	226142_at	-1.1476518	8.96232728	-5.2116592	6.57E-06	0.00744619	3.67864746
ECOP	238604_at	-1.1291845	9.93048271	-5.2069623	6.67E-06	0.00744619	3.66555947
CD40	205153_s_at	-1.1516665	8.96773222	-5.1878467	7.09E-06	0.00775153	3.61230284
IFIH1	219209_at	-1.1839872	8.4216604	-5.1481808	8.03E-06	0.0086134	3.50184783
IL4I1	230966_at	-1.2886142	10.7302425	-5.1035983	9.25E-06	0.00953971	3.3777992
MAFB	218559_s_at	-1.1661498	12.5070822	-5.0783026	1.00E-05	0.01013992	3.30746517
DAB2	201278_at	-1.1002534	9.40858428	-5.0704754	1.03E-05	0.01020405	3.28570957
P2RX4	204088_at	-1.11462	10.5804136	-5.0589302	1.06E-05	0.01039288	3.25362692
MLF1	204784_s_at	-1.1629248	9.99075341	-5.0382007	1.14E-05	0.01089897	3.19604331
STATH	206835_at	-1.2217631	10.6277925	-4.9970174	1.29E-05	0.01198544	3.08172746
GIMAP8	235306_at	-1.1769391	7.15724646	-4.9534429	1.48E-05	0.01329267	2.96090645
TATDN3	235069_at	-1.1552651	9.09091742	-4.9541849	1.48E-05	0.01329267	2.96296263
SYNJ2	216180_s_at	-1.1540719	8.13588041	-4.929194	1.60E-05	0.01372355	2.89373383
TREM1	219434_at	-1.1280034	11.1714599	-4.9272524	1.61E-05	0.01372355	2.88835722
ANXA2	213503_x_at	-1.1003875	13.1627385	-4.8992037	1.76E-05	0.01434614	2.81072248
G0S2	213524_s_at	-1.1355006	8.75639391	-4.864242	1.96E-05	0.01577044	2.71404776
IL10RA	204912_at	-1.1199911	10.5172379	-4.8323374	2.17E-05	0.01717268	2.62592271
BIRC3	210538_s_at	-1.257364	4.57445587	-4.8159991	2.28E-05	0.01765329	2.58083111
NT5E	203939_at	-1.2383789	8.45460557	-4.8143591	2.29E-05	0.01765329	2.57630644
CCL5	1555759_a_a	-1.0910656	13.6361191	-4.8032103	2.37E-05	0.01802488	2.54555373
SRPX2	205499_at	-1.224512	6.16138512	-4.767627	2.65E-05	0.01937153	2.4474847
ARL6IP5	200760_s_at	-1.1107109	11.7204466	-4.7591181	2.72E-05	0.01959427	2.42405329
EBI3	219424_at	-1.1775996	7.06372735	-4.7474062	2.82E-05	0.01976554	2.39181393
CD40	215346_at	-1.1705995	8.77095715	-4.7439006	2.86E-05	0.01976554	2.38216714
SRA1	224130_s_at	-1.094757	10.8027383	-4.7442703	2.85E-05	0.01976554	2.38318441
---	238501_at	-1.2105582	5.44953239	-4.73925	2.90E-05	0.01977223	2.36937124
SLIC1	229045_at	-1.1547886	8.27898536	-4.7357646	2.93E-05	0.01977223	2.35978293
C5ORF13	238411_x_at	-1.1683694	3.79974787	-4.7297145	2.98E-05	0.01979262	2.34314234
CARD15	220066_at	-1.1393689	8.86627619	-4.7275995	3.00E-05	0.01979262	2.33732587
PLEKHO1	218223_s_at	-1.1393641	9.19617144	-4.7232262	3.05E-05	0.01982503	2.32530094
NFE2L3	204702_s_at	-1.1949945	6.87002251	-4.7001908	3.27E-05	0.02069819	2.26199661
P2RY5	218589_at	-1.1719902	9.42743243	-4.6980902	3.29E-05	0.02069819	2.25622666
PSCDBP	209606_at	-1.1833853	8.14303401	-4.6940285	3.34E-05	0.02072301	2.24507189
PTX3	206157_at	-1.1323376	11.2731131	-4.6853648	3.43E-05	0.0210494	2.22128475
GHRL	223862_at	-1.184681	7.10722772	-4.6529266	3.79E-05	0.02252098	2.13230051
SGIP1	223672_at	-1.1713796	8.75400755	-4.6556052	3.76E-05	0.02252098	2.13964377
RPL13 /// LO	214976_at	-1.1498626	6.91302234	-4.6576183	3.73E-05	0.02252098	2.14516306
C13ORF31	1553141_at	-1.2240082	8.3027252	-4.6445617	3.89E-05	0.02286455	2.10937474
---	228573_at	-1.0954948	10.1938031	-4.631833	4.05E-05	0.02353205	2.07450537
---	213891_s_at	-1.1613505	8.36985954	-4.6072376	4.37E-05	0.02435882	2.00718586
C9ORF130	227893_at	-1.1466602	7.47771908	-4.6095058	4.34E-05	0.02435882	2.01339098
VPS33A	204590_x_at	-1.1717826	7.44063872	-4.5964894	4.51E-05	0.0246793	1.97779156
LACTB	1552486_s_at	-1.1146334	9.43978855	-4.5992016	4.48E-05	0.0246793	1.98520746
RAB27B	228708_at	-1.2146134	8.64471961	-4.5896791	4.61E-05	0.02495536	1.95917448
FXYD2	207434_s_at	-1.2391134	6.87935784	-4.5721048	4.87E-05	0.02609092	1.91116075
SOD2	216841_s_at	-1.1587002	8.29547849	-4.5601654	5.05E-05	0.02658149	1.87856531
INADL	214493_s_at	-1.1876014	5.13104061	-4.5382854	5.40E-05	0.02787086	1.81888217
BID	204493_at	-1.0962323	11.1430522	-4.5406927	5.36E-05	0.02787086	1.82544538
BTG1	240347_at	-1.1577464	6.46143817	-4.5117153	5.86E-05	0.02941834	1.74649575
CENTA2	219358_s_at	-1.1328376	10.4221094	-4.5125118	5.85E-05	0.02941834	1.74866433
LOC54103	213142_x_at	-1.1327449	10.7990817	-4.5010122	6.06E-05	0.02985684	1.71736561
---	232297_at	-1.1396483	8.4593218	-4.4977208	6.12E-05	0.02989176	1.7084108
THBD	203887_s_at	-1.0860181	12.8670409	-4.4922535	6.23E-05	0.02996581	1.6935397
KYNU	210663_s_at	-1.1273404	10.5827862	-4.4730592	6.61E-05	0.03124079	1.64136616
TATDN3	228867_at	-1.0979673	8.53193411	-4.4719767	6.63E-05	0.03124079	1.6384252
ITGB5	201125_s_at	-1.1305667	9.66682508	-4.4663279	6.74E-05	0.03151651	1.62308225

HNRPLL	225386_s_at	-1.088481	10.9979975	-4.4607622	6.86E-05	0.03178871	1.60796956
CRYBB2	206777_s_at	-1.1273748	9.09050704	-4.4486878	7.12E-05	0.03248173	1.57520029
CD55	1555950_a_a	-1.101176	11.0430169	-4.4455718	7.19E-05	0.03248173	1.56674717
MPEG1	226818_at	-1.102944	9.97050985	-4.4364675	7.39E-05	0.03312876	1.54205788
POPDC3	219926_at	-1.2923154	6.73431127	-4.4296067	7.55E-05	0.03333429	1.52346112
PLAUR	214866_at	-1.1197536	10.1465619	-4.4291541	7.56E-05	0.03333429	1.52223465
MGLL	239914_at	-1.1755243	5.02263039	-4.4192631	7.79E-05	0.03354952	1.49543825
EBI2	205419_at	-1.1647466	10.3787766	-4.4209004	7.75E-05	0.03354952	1.49987275
TPSAB1 /// T	207741_x_at	-1.154419	7.49088254	-4.4208416	7.76E-05	0.03354952	1.49971361
SLAMF8	219386_s_at	-1.1604525	10.1187042	-4.4123213	7.96E-05	0.03370605	1.47664099
VSNL1	203797_at	-1.1465556	9.26432896	-4.4080282	8.07E-05	0.03370605	1.46501964
ICAM2	213620_s_at	-1.129714	7.80787761	-4.4083036	8.06E-05	0.03370605	1.46576519
KYNU	217388_s_at	-1.1268071	11.2241113	-4.4076297	8.08E-05	0.03370605	1.4639412
ARHGAP18	225166_at	-1.1854012	7.40195979	-4.3979297	8.32E-05	0.03445978	1.43769541
MITF	226066_at	-1.1771138	7.94053593	-4.3803753	8.78E-05	0.03578326	1.39023722
MXD1	226275_at	-1.1272655	8.58697195	-4.3782779	8.84E-05	0.03578326	1.38457024
---	241389_at	-1.1324551	7.7082004	-4.3740983	8.95E-05	0.03597723	1.37327956
EGF	206254_at	-1.2273072	5.23328013	-4.3445051	9.80E-05	0.03909529	1.29342221
MALT1	210017_at	-1.1361187	7.85916694	-4.3417635	9.88E-05	0.03913824	1.28603152
AKR1C2	211653_x_at	-1.214586	9.21551731	-4.3261491	0.00010361	0.03933789	1.24396403
LPAAT-THET/	224480_s_at	-1.2033448	8.82402466	-4.3329062	0.00010149	0.03933789	1.26216342
MFI2	235911_at	-1.1178645	10.2132798	-4.3339031	0.00010118	0.03933789	1.26484915
ARHGAP18	225171_at	-1.1011604	10.7603262	-4.329986	0.0001024	0.03933789	1.25429744
TXNL2	209080_x_at	-1.0905642	11.1937357	-4.3267122	0.00010343	0.03933789	1.24548038
CD55	201926_s_at	-1.0876428	11.0124454	-4.3312016	0.00010202	0.03933789	1.25757168
ADAM9	202381_at	-1.1288776	10.068453	-4.2795249	0.00011941	0.04461757	1.11860933
OSBPL11	218304_s_at	-1.1200967	8.80861724	-4.268338	0.00012355	0.04461757	1.08859065
C5ORF32	224707_at	-1.1088424	9.60174121	-4.2676443	0.00012381	0.04461757	1.08672987
CD300A	209933_s_at	-1.0919093	11.068246	-4.2779818	0.00011998	0.04461757	1.11446708
ATP2C1	237278_x_at	-1.1252002	4.516837	-4.2604681	0.00012654	0.04474124	1.06748681
TGM5	207911_s_at	-1.0985729	10.9973837	-4.255458	0.00012848	0.04474124	1.05405768
CAST	208908_s_at	-1.0958388	10.4613552	-4.2557149	0.00012838	0.04474124	1.05474617
TNFRSF9	207536_s_at	-1.1669411	6.64800587	-4.2459826	0.00013222	0.04518347	1.02867283
HMGB2	243368_at	-1.1602025	4.88716386	-4.2463169	0.00013209	0.04518347	1.02956814
TNFAIP3	202644_s_at	-1.1253187	10.6490533	-4.2484635	0.00013123	0.04518347	1.03531746
C1ORF21	223125_s_at	-1.1101543	11.0658204	-4.2399019	0.00013469	0.04573895	1.01239127
---	212387_at	-1.148179	7.86470579	-4.2245512	0.0001411	0.04762227	0.97132047

12 hr:

Gene	ID	raw.FC	AveExpr	t	P.Value	adj.P.Val	B
PRDM1	228964_at	-1.3131187	7.67111264	-8.6246024	1.41E-10	7.72E-06	11.3048141
RCSD1	239328_at	-1.2917804	9.91539671	-6.452108	1.22E-07	0.0033307	6.31495199
BTG1	240347_at	-1.2562187	6.63516956	-5.7047112	1.33E-06	0.02422499	4.46209908
FBXO45	225099_at	1.13350791	9.69247626	5.51958363	2.40E-06	0.03282196	3.99779146
ZNF566	240239_at	1.20902406	6.20206745	5.35216774	4.09E-06	0.04477351	3.57707114

24 hr:

Gene	ID	raw.FC	AveExpr	t	P.Value	adj.P.Val	B
MMP9	203936_s_at	-1.2587837	8.76550502	-6.6308842	7.89E-08	0.00431641	6.0372842

Downregulated:

6 hr:

Gene	ID	raw.FC	AveExpr	t	P.Value	adj.P.Val	B
LOC648342	1569392_at	1.25828385	6.46339373	5.71764607	1.32E-06	0.00281664	5.09166798
BTBD14A	243431_at	1.31196093	8.69387594	5.37680876	3.90E-06	0.00508408	4.13936456
SERPINB2	204614_at	1.25751853	10.7388922	5.14040426	8.23E-06	0.00865761	3.48020227
MYB	204798_at	1.09897729	13.1513017	5.00361266	1.27E-05	0.01194194	3.10002645
STT3B	238303_at	1.2645975	6.00460112	4.9254338	1.62E-05	0.01372355	2.88332163
HSPD1	241716_at	1.30689927	6.02121553	4.9223221	1.64E-05	0.01372355	2.87470616
ACTN4	232058_at	1.19228742	8.60657642	4.91815847	1.66E-05	0.01372355	2.86317939
FAM62A	244234_at	1.14840119	7.29749849	4.78192868	2.54E-05	0.01899888	2.48688527
HIC2	1559600_at	1.17980654	8.38342055	4.76703429	2.66E-05	0.01937153	2.4458524
OXCT2	235275_at	1.16642629	12.3300201	4.71420617	3.13E-05	0.02014951	2.30050545
---	228907_at	1.17599277	8.21884465	4.6120071	4.30E-05	0.02435882	2.02023432
GAPDHS	222280_at	1.1646993	7.71035699	4.60723903	4.37E-05	0.02435882	2.00718978
SPFH1	202441_at	1.12657525	11.0883546	4.55979394	5.06E-05	0.02658149	1.87755147
NAPB	1570441_at	1.20267749	5.92073389	4.52611168	5.61E-05	0.02866712	1.78570418
HNRPD	213359_at	1.16888386	9.72519123	4.50340998	6.02E-05	0.02985684	1.72389004
CISH	223377_x_at	1.09133621	9.01105134	4.49116936	6.25E-05	0.02996581	1.69059129
SFRS14	64371_at	1.12400512	8.1962891	4.44643142	7.17E-05	0.03248173	1.5690791
C14ORF10	239188_at	1.16314645	6.54891766	4.38855767	8.56E-05	0.03519643	1.4123519
---	232903_at	1.21778341	6.93050721	4.29620676	0.0001135	0.04279881	1.16341547
HDAC4	232225_at	1.14445571	7.77327378	4.27489999	0.00012111	0.04461757	1.10619611
CLK1	214683_s_at	1.160507	9.27028645	4.268656	0.00012343	0.04461757	1.08944366
CRLF3	235803_at	1.19940674	9.07113133	4.2670238	0.00012404	0.04461757	1.08506571
ZNF250	241738_at	1.11389598	4.2390353	4.26401964	0.00012518	0.04473242	1.07700912
WDFY1	234157_at	1.20859908	6.39798409	4.25895039	0.00012712	0.04474124	1.06341818

12 hr:

Gene	ID	raw.FC	AveExpr	t	P.Value	adj.P.Val	B
FBXO45	225099_at	1.13350791	9.69247626	5.51958363	2.40E-06	0.03282196	3.99779146
ZNF566	240239_at	1.20902406	6.20206745	5.35216774	4.09E-06	0.04477351	3.57707114

SUPPLEMENTARY TABLE 4a

Pathways significantly enriched among genes upregulated by Probioglat relative to GA (mannitol-corrected comparison) at 6 hours:

Term	Pvalue	Fold Enrichm	Benjamini
GO:0005576~extracellular region	1.59E-09	4.04256216	2.49E-07
GO:0002376~immune system process	1.04E-08	4.15662651	1.53E-05
GO:0048583~regulation of response to stimulus	1.56E-07	5.89709585	0.00011513
GO:0044421~extracellular region part	2.87E-06	4.6644948	0.00022533
GO:0048519~negative regulation of biological process	6.21E-07	2.62528519	0.00030526
GO:0006955~immune response	1.10E-06	4.64403067	0.0003252
GO:0002237~response to molecule of bacterial origin	1.01E-06	14.5326132	0.00037043
GO:0005886~plasma membrane	7.17E-06	2.13272566	0.00037532
GO:0051707~response to other organism	2.07E-06	7.28915663	0.00050768
GO:0050878~regulation of body fluid levels	2.72E-06	12.5804711	0.00057207
GO:0032879~regulation of localization	3.74E-06	4.5284289	0.00068862
GO:0032496~response to lipopolysaccharide	6.46E-06	14.7506024	0.00086508
GO:0060089~molecular transducer activity	5.73E-06	2.80749625	0.00087605
GO:0004871~signal transducer activity	5.73E-06	2.80749625	0.00087605
GO:0065007~biological regulation	5.36E-06	1.47735835	0.00087776
GO:0065008~regulation of biological quality	6.39E-06	2.87122423	0.00094178
GO:0050789~regulation of biological process	7.87E-06	1.49362031	0.0009662
GO:0051049~regulation of transport	8.87E-06	5.49711891	0.00100533
GO:0004872~receptor activity	4.04E-06	3.35789983	0.00123687
GO:0009611~response to wounding	1.27E-05	4.78915663	0.00124947
hsa04060:Cytokine-cytokine receptor interaction	1.73E-05	6.21238938	0.00127682
GO:0002682~regulation of immune system process	1.23E-05	5.3123418	0.00129048
GO:0048518~positive regulation of biological process	1.62E-05	2.29046621	0.00132631

GO:0051239~regulation of multicellular organismal process	1.72E-05	3.49832925	0.00133056
GO:0060341~regulation of cellular localization	1.61E-05	7.83680175	0.00139875
GO:0051046~regulation of secretion	1.57E-05	9.68840881	0.0014461
GO:0002822~regulation of adaptive immune response based on somatic recombination of immune receptors built from immunoglobulin superfamily domains	3.26E-05	15.8042169	0.00184666
GO:0043067~regulation of programmed cell death	2.93E-05	3.18205709	0.00187738
GO:0010941~regulation of cell death	3.06E-05	3.17141476	0.00187808
GO:0009617~response to bacterium	3.20E-05	8.68960378	0.00188684
GO:0042981~regulation of apoptosis	2.57E-05	3.21441699	0.00189579
GO:0050776~regulation of immune response	2.88E-05	7.23857261	0.00192627
GO:0048513~organ development	2.80E-05	2.61696896	0.0019623
GO:0002819~regulation of adaptive immune response	3.69E-05	15.4187482	0.00201028
GO:0009605~response to external stimulus	4.27E-05	3.43336687	0.00224359
GO:0045428~regulation of nitric oxide biosynthetic process	4.62E-05	23.9457831	0.00234521
GO:0051223~regulation of protein transport	5.39E-05	10.2434739	0.00264462
GO:0009607~response to biotic stimulus	5.71E-05	4.99558579	0.00270962
GO:0005615~extracellular space	7.88E-05	4.83163919	0.00308733
GO:0051241~negative regulation of multicellular organismal process	7.32E-05	9.70434369	0.0033662
GO:0032101~regulation of response to external stimulus	8.47E-05	9.45551437	0.00366669
GO:0070201~regulation of establishment of protein localization	8.47E-05	9.45551437	0.00366669
GO:0050793~regulation of developmental process	8.33E-05	3.66930408	0.00371381
GO:0048522~positive regulation of cellular process	9.67E-05	2.22534329	0.00406592
GO:0032501~multicellular organismal process	0.000104285	1.86057044	0.00426099
GO:0007166~cell surface receptor linked signal transduction	0.000113804	2.85618377	0.0045237

GO:0006916~anti-apoptosis	0.000124449	5.88977026	0.00481596
GO:0043066~negative regulation of apoptosis	0.000129722	4.52724962	0.00489113
GO:0060548~negative regulation of cell death	0.000147362	4.45759963	0.00515874
GO:0043069~negative regulation of programmed cell death	0.000142772	4.47481044	0.00524771
GO:0009893~positive regulation of metabolic process	0.000147162	2.92670683	0.00527708
GO:0031347~regulation of defense response	0.000165947	8.3810241	0.00567284
GO:0006952~defense response	0.000177009	3.96343997	0.00591277
GO:0050708~regulation of protein secretion	0.000186347	16.9937816	0.00608588
GO:0051704~multi-organism process	0.000215223	3.33724036	0.00672774
GO:0032880~regulation of protein localization	0.00021195	8.01663174	0.00676933
GO:0002697~regulation of immune effector process	0.000233713	10.5361446	0.00700664
GO:0002706~regulation of lymphocyte mediated immunity	0.000238915	15.9638554	0.00701932
GO:0080134~regulation of response to stress	0.000230106	5.3878012	0.00704207
GO:0032502~developmental process	0.000273185	1.8912593	0.00786556
GO:0048584~positive regulation of response to stimulus	0.000283953	6.15249318	0.00801779
GO:0007155~cell adhesion	0.000307737	3.71863926	0.00852335
GO:0022610~biological adhesion	0.000315593	3.70773416	0.00857888
GO:0002684~positive regulation of immune system process	0.000324184	6.02065404	0.00865189
GO:0001775~cell activation	0.000386538	4.99080533	0.01012459
GO:0045429~positive regulation of nitric oxide biosynthetic process	0.000407508	26.3403614	0.0104848
GO:0002703~regulation of leukocyte mediated immunity	0.00041591	13.8633481	0.01051636
GO:0048856~anatomical structure development	0.000458417	2.01871598	0.01138993
GO:0046903~secretion	0.000554725	5.5090952	0.01353905
GO:0050727~regulation of inflammatory response	0.000612986	12.5430293	0.01470747
GO:0048731~system development	0.000635394	2.0659107	0.0149972

GO:0031226~intrinsic to plasma membrane	0.000511383	2.90140135	0.01593322
GO:0051051~negative regulation of transport	0.00074669	8.20998279	0.01732488
GO:0051050~positive regulation of transport	0.000772048	6.30367624	0.01763083
GO:0051384~response to glucocorticoid stimulus	0.000868651	11.4523311	0.01951397
GO:0005625~soluble fraction	0.000768008	4.51789639	0.0199032
GO:0031325~positive regulation of cellular metabolic process	0.000976424	2.70157553	0.02158118
GO:0050817~coagulation	0.001021169	10.9751506	0.02222647
GO:0007596~blood coagulation	0.001021169	10.9751506	0.02222647
GO:0031960~response to corticosteroid stimulus	0.001104078	10.7511679	0.02366131
GO:0051047~positive regulation of secretion	0.001104078	10.7511679	0.02366131
GO:0030097~hemopoiesis	0.001226255	4.81652324	0.02515165
GO:0007275~multicellular organismal development	0.001212098	1.85689232	0.02521544
GO:0010033~response to organic substance	0.001204931	2.95193706	0.02542679
hsa04621:NOD-like receptor signaling pathway	0.000725707	7.94716981	0.02650336
GO:0002252~immune effector process	0.00136637	7.18373494	0.02760362
GO:0032680~regulation of tumor necrosis factor production	0.001395324	17.560241	0.02780002
GO:0006954~inflammatory response	0.001443579	4.68273092	0.02836525
GO:0007599~hemostasis	0.001483225	9.93975904	0.02875056
GO:0051173~positive regulation of nitrogen compound metabolic process	0.001639177	3.03927247	0.03014551
GO:0051240~positive regulation of multicellular organismal process	0.001622601	5.46318608	0.03022181
GO:0042127~regulation of cell proliferation	0.00158235	2.85949644	0.0302467
GO:0005887~integral to plasma membrane	0.001373673	2.75859094	0.03036028
GO:0050896~response to stimulus	0.0016107	1.7338691	0.03038701
GO:0002700~regulation of production of molecular mediator of immune response	0.001768391	16.2094532	0.03208543
GO:0042113~B cell activation	0.001821278	9.40727194	0.03262879
GO:0007165~signal transduction	0.001988349	1.86414448	0.03472894

GO:0048534~hemopoietic or lymphoid organ development	0.001967993	4.4362714	0.0347911
GO:0005102~receptor binding	0.000373426	3.37939364	0.03738004
GO:0050794~regulation of cellular process	0.002340559	1.34948203	0.04028545
GO:0046651~lymphocyte proliferation	0.002435526	14.5326132	0.04140481
GO:0070661~leukocyte proliferation	0.002687978	14.0481928	0.04508478
GO:0032943~mononuclear cell proliferation	0.002687978	14.0481928	0.04508478
GO:0002520~immune system development	0.002858408	4.1521752	0.04734076
GO:0042994~cytoplasmic sequestering of transcription factor	0.002996182	35.1204819	0.04901917
GO:0042100~B cell proliferation	0.002996182	35.1204819	0.04901917

SUPPLEMENTARY TABLE 4b

Pathways significantly enriched among genes upregulated by Probioglat relative to GA (mannitol-corrected comparison) at 6 hours, earlier run including Kegg and GO BP only:

Pathway	Fold Enrichment	PValue	Benjamini
Cytokine-cytokine receptor interaction	4.35698707	1.27E-04	0.00976734
NOD-like receptor signaling pathway	10.04279131	2.62E-04	0.01003313
response to molecule of bacterial origin	13.53138285	1.85E-06	0.00274795
immune response	3.794670407	3.41E-06	0.00253001
regulation of apoptosis	3.437543465	6.44E-06	0.00318073
regulation of programmed cell death	3.403676042	7.38E-06	0.00273572
regulation of cell death	3.391147173	7.77E-06	0.00230315
response to wounding	4.116859404	1.28E-05	0.00315938
response to lipopolysaccharide	13.22385142	1.35E-05	0.0028572
regulation of nitric oxide biosynthetic process	26.93747511	3.12E-05	0.00576833
positive regulation of immune system process	6.1118641	3.39E-05	0.0055742
regulation of adaptive immune response based on somatic recombination of immune receptors built from immunoglobulin superfamily domains	15.8686217	3.47E-05	0.00513317

regulation of adaptive immune response	15.58525346	3.79E-05	0.00509678
regulation of cellular localization	5.865417967	4.67E-05	0.00576377
regulation of body fluid levels	8.253183863	4.82E-05	0.00548643
defense response	3.547862575	6.58E-05	0.00694762
regulation of secretion	6.480996487	6.85E-05	0.00675163
regulation of cell proliferation	3.142134962	7.26E-05	0.00671416
anti-apoptosis	6.355151895	7.86E-05	0.00683534
regulation of protein transport	8.931899642	1.24E-04	0.01018322
negative regulation of apoptosis	4.52001701	1.42E-04	0.01104867
cell activation	5.068375108	1.43E-04	0.01055706
negative regulation of programmed cell death	4.457064127	1.59E-04	0.01120592
negative regulation of cell death	4.444683393	1.63E-04	0.01094271
B cell activation	11.48387097	1.65E-04	0.01056607
regulation of establishment of protein localization	8.415178175	1.72E-04	0.01059381
secretion	4.84874552	1.99E-04	0.0117664
response to bacterium	6.029528107	3.40E-04	0.01921489
response to organic substance	3.026262807	3.46E-04	0.01885962
regulation of protein localization	7.378525791	3.51E-04	0.01843931
inflammatory response	4.475765095	3.60E-04	0.01826237
positive regulation of nitric oxide biosynthetic process	27.70711726	3.70E-04	0.01816152
regulation of lymphocyte mediated immunity	13.46873755	4.89E-04	0.02311946
regulation of lymphocyte activation	6.879976751	5.10E-04	0.02337339
positive regulation of lymphocyte activation	8.997672098	5.13E-04	0.022829
regulation of immune effector process	8.641328649	6.18E-04	0.02660928
regulation of protein secretion	12.5398591	6.42E-04	0.02685938
regulation of response to external stimulus	6.404003517	7.44E-04	0.03021027
positive regulation of leukocyte activation	8.233718807	7.70E-04	0.03040558
regulation of leukocyte mediated immunity	11.92314472	7.77E-04	0.02991572
positive regulation of transport	5.218380828	8.05E-04	0.03016938
cell adhesion	2.909247312	8.70E-04	0.03175969
positive regulation of secretion	8.007102693	8.73E-04	0.03112191
negative regulation of multicellular organismal process	6.208759507	8.74E-04	0.03043604
biological adhesion	2.905097173	8.81E-04	0.02996299
regulation of B cell proliferation	20.78033794	8.82E-04	0.02930901

regulation of leukocyte activation	6.133955176	9.31E-04	0.03025871
positive regulation of cell activation	7.862830573	9.48E-04	0.03012533
positive regulation of response to stimulus	4.930927647	0.00112022	0.03477139
hemopoiesis	4.930927647	0.00112022	0.03477139
regulation of tumor necrosis factor production	18.7693375	0.00119195	0.03620158
regulation of cell activation	5.818494624	0.00122343	0.03639613
leukocyte activation	4.808673243	0.00129524	0.03773715
positive regulation of nitrogen compound metabolic process	2.9363521	0.00135277	0.0386239
positive regulation of multicellular organismal process	4.769257888	0.00135811	0.03804226
regulation of cytokine production	5.625616349	0.00145409	0.03992524
regulation of inflammatory response	9.569892473	0.00176885	0.04748898
positive regulation of cell communication	3.979213648	0.00177694	0.04685454
adaptive immune response	9.445608155	0.00185618	0.048042
adaptive immune response based on somatic recombination of immune receptors built from immunoglobulin superfamily domains	9.445608155	0.00185618	0.048042
response to glucocorticoid stimulus	9.324510615	0.00194645	0.04946032
hemopoietic or lymphoid organ development	4.475765095	0.00195153	0.04875256
response to steroid hormone stimulus	5.303315412	0.00196163	0.04818865
humoral immune response	9.206478835	0.00203972	0.04924664

SUPPLEMENTARY TABLE 5

Reported Adverse Events and Relapses in Teva's Patient Support Program, 2012-2013

	**2012** (Copaxone alone)	**2013** (Probioglat+ Copaxone)	**Statistical Significance of Increase** from 2012 to 2013
Adverse Events	125	380	p < 2.2e-29*
Adverse Event Rate (per month)	10.4	31.7	
Relapses	8	59	p < 3e-11*
Relapse Rate (per month)	0.7	4.9	
Total Number of Patients in Teva's Patient Support Program	1618	1552	

* Single tailed p-value by Fisher's Exact test comparing the number of adverse events and relapses in 2012 < 2013.

SUPPLEMENTARY TABLE 6

Table of Probioglat expression values for key genes compared to the range set by observed expression values under Copaxone treatment:

Red text indicates that the expression value for this Probioglat treated sample is outside of the observed range of all Copaxone samples.

		Copaxone Expression Value Range		Probioglat Expression Values					
Probeset	Gene	Maxi-mum	Mini-mum	Sample 1	Sample 2	Sample 3	Sample 4	Sample 5	Sample 6
203936_s_at	MMP9	9.97	9.53	10.14	10.05	10.12	10.14	10.04	10.04
201743_at	CD14	9.69	9.32	9.75	9.75	9.67	9.79	9.71	9.86
202637_s_at	ICAM1	8.06	7.44	8.14	7.96	8.26	7.84	8.18	7.92
202638_s_at	ICAM1	7.30	6.51	7.67	7.54	7.45	7.32	7.19	6.94
223961_s_at	CISH	7.69	7.24	7.31	7.20	7.41	7.37	7.62	7.23
223377_x_at	CISH	9.11	8.86	8.81	8.81	8.82	8.85	8.84	8.88
